# A fuzzy ZE-number group decision-making framework using BWM and MABAC for risk assessment in medicinal plant extraction

**DOI:** 10.1371/journal.pone.0342976

**Published:** 2026-02-24

**Authors:** Fatemeh Gheytasi, Masoumeh Kianifard, Saeid Jafarzadeh Ghoushchi, Salar Hafez-Ghoran, Mohammad Reza Maghami, Mazlan Mohamed

**Affiliations:** 1 Department Pharmaceutical Technology, Faculty of Pharmacy, University Malaya, Malaysia; 2 Department Industrial Engineering, Urmia University of Technology, Urmia, Iran; 3 Laboratory for Functional Foods and Human Health, Center for Excellence in Post-Harvest Technologies, North Carolina Agricultural and Technical State University, North Carolina Research Campus, 500 Laureate Way, Kannapolis, North Carolina, United States; 4 Strategic Research Institute (SRI), Asia Pacific University of Technology and Innovation, (APU), Kuala Lumpur, Malaysia; 5 Faculty of Artificial Intelligence and Cyber Security (FAIX), Universiti Teknikal Malaysia Melaka, Tunggal, Malaysia; Istanbul University: Istanbul Universitesi, TÜRKIYE

## Abstract

The extraction of bioactive compounds from medicinal plants is a crucial process in pharmaceutical and herbal medicine industries, yet it presents numerous challenges that can compromise quality, efficiency, and standardization. This study integrates Failure Mode and Effects Analysis (FMEA) with an advanced multi-criteria decision-making framework based on ZE-numbers, employing the ZE-based Best–Worst Method (ZE-BWM) to determine the relative importance of risk criteria and the ZE-MABAC method to rank the most critical risks in medicinal plant extraction. By incorporating decision makers’ assessments, fuzzy logic, and reliability factors, this study enhances traditional risk assessment approaches by accounting for uncertainty and expert reliability, leading to a more precise decision-making framework. The findings indicate that sample contamination, improper handling, suboptimal extraction conditions, and instrumental calibration errors are the most significant failure modes affecting extraction efficiency and product quality. Sample-related risks, such as inconsistencies in raw material quality and contamination, can lead to variability in bioactive compound concentrations. Similarly, inefficient extraction techniques and poor solvent selection impact the purity and yield of the final product, while instrumental errors and inadequate calibration introduce measurement inconsistencies. To mitigate these risks, process standardization, rigorous quality control, and personnel training are essential. Additionally, integrating real-time monitoring systems, sustainable extraction techniques, and AI-driven predictive models can further enhance extraction reliability and efficiency. This research advances risk assessment methodologies by offering a structured and data-driven framework for optimizing medicinal plant extraction, ensuring consistency, and improving product quality. Future studies should focus on automation, real-time risk monitoring, and green extraction technologies to further enhance extraction processes.

## 1. Introduction

The extraction of bioactive compounds from medicinal plants is a crucial process in developing therapeutic products. However, it is often met with various barriers and risks that can impact the quality, consistency, and efficacy of the final extract. One of the most common challenges is the chemical variability of plant materials. Factors such as plant species, environmental conditions, harvest time, and the specific plant part used for extraction can all influence the chemical composition [1,2]. This variability leads to inconsistent yields and potencies of active compounds, making it difficult to standardize the extraction process. Moreover, issues such as improper handling or poor-quality raw materials can introduce contaminants, further compromising the quality of the extract [3]. The extraction method itself also presents a significant risk. There are several techniques available—such as solvent extraction, steam distillation, and cold pressing—each with its own strengths and weaknesses. Some methods, like solvent extraction, may leave behind harmful residual solvents, while others, such as high-heat methods, could degrade sensitive compounds [4]. Choosing the right extraction method is critical for optimizing yield, purity, and safety. Missteps in this choice can lead to poor-quality extracts, making it important to assess the risks and select the most suitable method for each plant species and its specific bioactive compounds.

One effective way to manage these risks is through the use of Failure Mode and Effect Analysis (FMEA). It is a structured approach widely used across industries like pharmaceuticals, automotive, aerospace, and chemical manufacturing to identify, evaluate, and mitigate potential failures in complex processes [5,6]. Researchers in these fields have applied FMEA to analyze every step of production to detect potential failures, assess their consequences, and prioritize corrective actions. This methodology can be similarly applied to medicinal plant extraction to identify critical failure points, such as contamination during handling, variability in raw materials, or inefficiencies in the extraction method. By conducting FMEA on the plant extraction process, researchers can systematically evaluate each stage for possible failure modes and their potential impact on the quality and safety of the final product [7]. For example, FMEA can help determine how different extraction methods might affect the concentration of active compounds, identify points where contamination might occur, or highlight where chemical variability may lead to inconsistent results. Using FMEA in the extraction process allows for a more controlled and optimized approach, ensuring that risks are mitigated and the final product is of consistent quality. Ultimately, adopting this method can improve the overall safety, efficacy, and standardization of medicinal plant extracts.

FMEA is a widely used risk assessment tool across various industries, particularly in the automotive and manufacturing sectors [8]. It aims to identify and mitigate potential failures in systems, processes, designs, or services [9] While FMEA has proven effective in reducing medical errors and potential failure rates in healthcare settings [10]. The conventional Risk Priority Number (RPN) method has been criticized for its limitations. Researchers have proposed various risk priority models to enhance FMEA performance, focusing on addressing shortcomings, exploring popular approaches, and identifying inadequacies [9]. The evolution of FMEA techniques has led to the development of different categories, including FMEA types, formats, implemented subjects, and automation. Future research should consider applying different analysis approaches based on FMEA implementation objectives and developing concrete procedures for System, Design, and Process FMEA.

Rustandi et al. [11] examine the application of FMEA in the production of Spirulina capsules within Indonesia’s traditional medicine sector. Conducted by Rustandi and collaborators, the research emphasizes the necessity of quality risk management to ensure consumer safety and compliance with Indonesian Food and Drug Supervisory Agency (BPOM) standards. Utilizing FMEA and fishbone analysis, the study identifies and evaluates potential risks at critical production stages, including receiving raw materials, storage, mixing, filling, and final storage. Factors identified as errors include substandard raw material quality, microbial contamination, heavy metal contamination, inadequate environmental controls, and failures in visual inspections. The assessment calculates RPNs based on severity (S), occurrence (O), and detectability (D). Key risks such as microbial and heavy metal contamination are highlighted, with recommended controls focusing on raw material verification, optimal storage conditions, and monitoring moisture and microbial levels. The findings demonstrate that implementing FMEA significantly enhances product quality and safety, serving as a valuable model for improving risk management in traditional medicine manufacturing.

Feili et al. [12] aims to enhance the safety and efficiency of solar drying methods for herbal plants by identifying and mitigating potential risks throughout the drying process. It focuses on various failure modes, including biological contamination due to pathogens, chemical residues from pesticides and heavy metals, and physical hazards like foreign matter integration. The study employs FMEA and Hazard Analysis and Critical Control Points (HACCP) as risk assessment techniques. Findings indicate that critical control points in the drying process significantly affect product quality, with error variables such as improper temperature control, inadequate drying time, and insufficient hygiene practices flagged as major risks. By systematically analyzing these failure modes, the paper underscores the importance of monitoring critical limits and implementing corrective actions. The integration of FMEA with HACCP resulted in substantial reductions in RPNs, demonstrating improved safety and quality assurance in solar drying systems. Overall, this approach provides valuable insights for optimizing the herbal plants drying industry while promoting the use of renewable energy.

Hajimolaali et al. [13] aimed to identify and evaluate quality risks in the pharmaceutical industry using a combined approach of FMEA and Fuzzy TOPSIS methods [14]. Researchers identified over 100 potential risks, ultimately narrowing it down to 20 critical quality-related risks, such as human errors in production, inadequate supervision of machinery qualification, and improper handling of non-conforming products. The analysis revealed that the significant risk factors stemmed largely from quality assurance practices, highlighting the need for enhanced focus to mitigate these risks effectively. Overall, the findings underscored the critical role of quality management in ensuring pharmaceutical safety and efficacy. Ismael et al. [15] focus on implementing Quality Risk Management (QRM) within Al-Hokamaa Pharmaceutical Company, aiming to identify and mitigate risks associated with drug production processes using FMEA as the primary technique. Key risk areas identified include errors in manufacturing stages such as Parting, Pressing, Coating, and Preparation. The objective is to calculate the RPN of potential failures on a scale of 1–10, where 10 indicates the highest risk. Errors considered for calculating RPN include inadequate quality control and machine malfunctions. The findings revealed significant risk factors, such as poor raw material quality and inadequate training of personnel, which led to a notable reduction in RPN from 8442 to 5253, indicating improved risk management and process quality. Rustandi et al. [11] apply FMEA to evaluate risks in Spirulina capsule production in Indonesia. The aim is to enhance product quality and safety by identifying error risks such as substandard raw materials and uncontrolled storage conditions. The risk evaluation technique employs fishbone analysis to categorize potential causes. RPN is calculated, and key error variables include raw material quality, environmental controls, and inspection methods. Findings indicate that risks like substandard materials (RPN = 336) and poor storage conditions (RPN = 420) require immediate control measures to mitigate consumer safety risks.

In addition to pharmaceutical and herbal applications, fuzzy FMEA and its hybrid extensions have been increasingly employed in other high-risk and complex engineering domains. Recent studies have integrated fuzzy FMEA with advanced multi-criteria decision-making techniques, such as DEMATEL, entropy-based weighting, and evidence theory, to better capture interdependencies among risk factors and reduce subjectivity in risk prioritization.

Notably, the maritime domain has witnessed a growing body of research applying fuzzy FMEA and FMECA to assess the reliability and safety of shipboard systems. For example, Ceylan [16] investigated turbocharger fouling risks in marine diesel engines using fuzzy FMEA, while Karanović et al. [17] proposed a fuzzy FMEA-based framework for risk analysis of hydraulic steering systems. Similarly, Sezer et al. [18] developed a Dempster-Shafer evidence-based FMECA approach for ballast water systems, and Ceylan [19] applied rule-based fuzzy FMEA to evaluate compressor system risks for preventing major marine accidents. In recent years, fuzzy ZE-numbers have attracted increasing attention in group decision-making research due to their ability to simultaneously capture uncertainty, expert reliability, and consensus. Several studies have successfully applied fuzzy ZE-numbers to complex decision problems in different domains. For instance, Salteh et al. [20] employed a fuzzy ZE-number framework to evaluate and prioritize barriers to eco-regenerative supply chain implementation, while Haseli et al. [21] applied fuzzy ZE-numbers within a group decision-making framework for the selection of smart jewelry in female technology applications.

More recently, fuzzy ZE-numbers have been integrated with advanced multi-criteria decision-making methods to address sustainability and resilience-oriented problems. Ecer et al. [22] proposed a combined framework under fuzzy ZE-numbers for evaluating sustainable cold chain suppliers, and Haseli et al. [23] developed a ZE-number-based decision-making model to support climate-resilient land-use and transportation projects. These studies demonstrate the effectiveness of fuzzy ZE-numbers in providing a reliable and structured representation of expert judgments in complex and uncertain environments. Building upon these existing developments, the present study adopts fuzzy ZE-numbers as a decision-support mechanism rather than proposing a new theoretical extension. The contribution of this research lies in integrating fuzzy ZE-numbers with FMEA, ZE-BWM, and ZE-MABAC to construct a coherent and application-oriented risk assessment framework tailored to medicinal plant extraction processes.

Among various multi-criteria decision-making methods, MABAC was selected in this study due to its ability to provide a stable and interpretable ranking of alternatives based on their distance from a border approximation area. This feature is particularly compatible with FMEA-based risk assessment, where the objective is to prioritize failure modes according to their relative criticality. Moreover, MABAC has a simple computational structure and can be efficiently integrated with fuzzy and ZE-number-based information representations, enabling effective handling of uncertainty and expert reliability without increasing methodological complexity. ZE-numbers were adopted in this study to enhance the representation of expert evaluations by simultaneously modeling uncertainty, reliability, and group consensus. Unlike conventional fuzzy numbers, which only address uncertainty, and Z-numbers, which incorporate an individual reliability component, ZE-numbers explicitly account for collective agreement, disagreement, and neutrality among experts. This capability is particularly valuable in FMEA-based risk assessment of medicinal plant extraction processes, where heterogeneous expert opinions and varying confidence levels can significantly influence risk prioritization outcomes.

### 1.1. Critical Barriers in Medical Plant Extraction Identification

Medicinal plant extraction generally follows a series of common steps, regardless of plant species or specific extraction techniques. These steps typically include sample preparation, drying, grinding, solvent selection, extraction, and concentration of the resulting extract. Although various methods such as maceration, Soxhlet extraction, ultrasound-assisted extraction, and solvent extraction differ in their operational details, they share similar challenges, including solvent efficiency, heat sensitivity, extraction time, and potential loss of bioactive compounds. Because these issues occur across a wide range of plants and extraction methods, this study evaluates extraction challenges from a broad and general perspective rather than focusing on a single plant or technique. The process of extracting medicinal compounds from plants is intricate and highly sensitive to various risks that can affect the final product’s quality and consistency. Sample-related risks are among the most common sources of error in plant extract analysis. One significant challenge is the insufficient sample amount or quality, which can result in an incomplete representation of the plant’s bioactive compounds, leading to inaccurate results. Inappropriate sample preparation techniques also pose a risk, as improper handling, grinding, or storage of plant material can degrade or alter the compounds of interest. Additionally, contamination during sample collection or handling is a prevalent issue, as even small amounts of foreign substances can compromise the integrity of the sample. The freshness of the plant material plays a crucial role as well, as older or poorly stored plant samples may yield lower metabolite quantities and affect the stability of the compounds during extraction. Another significant source of risk is related to the extraction method used. One of the primary concerns is the inadequate extraction solvent or concentration. Choosing the wrong solvent or the incorrect concentration can result in incomplete or inefficient extraction of the bioactive compounds [4]. Similarly, suboptimal extraction conditions, such as inappropriate time, temperature, or pH, can hinder the extraction process, leading to low yields or degradation of sensitive compounds [24]. Inefficient extraction techniques, like improper use of solid-liquid or liquid-liquid extraction methods, can also be a major concern if they do not adequately break down plant materials or extract the desired metabolites. Moreover, some extraction methods may be incompatible with the target metabolites, affecting the purity or completeness of the extraction. These issues underscore the importance of choosing the right solvent and extraction conditions to optimize the yield and quality of the plant extract. Instrumental calibration and reference standards also introduce risks during the analysis phase. Inaccurate or malfunctioning equipment can result in unreliable data, affecting the precision and reliability of the results. Poor calibration or incorrect settings on analytical instruments such as spectrometers or chromatographs can lead to faulty measurements or incorrect identification of metabolites. Contamination in the analytical system can also alter the results, leading to false positives or negatives. Furthermore, instrumental and device errors, such as inconsistent temperature control or pressure in chromatographic systems, can influence the accuracy of measurements, making it essential to regularly maintain and calibrate equipment to avoid these issues.

Analytical method-related issues are critical factors to consider in risk analysis as well. One of the most common problems is the inappropriate choice of analytical technique, such as selecting the wrong chromatography or spectroscopy method for the plant extract. Each analytical technique has its strengths and limitations, and improper selection can result in inadequate detection or quantification of target metabolites. Furthermore, some methods may lack the necessary specificity or sensitivity, making it difficult to identify trace compounds or detect metabolites at low concentrations. Inadequate separation or detection of target metabolites can result in overlapping peaks or incorrect identification, reducing the method’s accuracy. Additionally, matrix interference, where other components of the plant extract interfere with the analysis, is a common challenge, as well as the lack of reference standards for identifying and quantifying metabolites. Human-related factors also play a significant role in the risk analysis process. Operator-related issues can be a major source of error if the personnel conducting the extraction or analysis are not sufficiently trained or experienced. Inconsistent or improper execution of the extraction method due to a lack of expertise can lead to variability in the results. Moreover, human errors during sample handling, such as incorrect labeling, improper storage, or contamination, can compromise the integrity of the entire process [25]. Even after extraction, errors in data processing or inadequate statistical analysis can affect the interpretation of the results, leading to incorrect conclusions or missed insights. Proper training and quality control measures are essential to minimize these risks and ensure consistency in the process.

Finally, biological and environmental factors can significantly impact the outcome of medicinal plant extraction [26]. One such risk is the variability in metabolite content due to factors like plant species, genotype, and developmental stage. These natural differences can make it difficult to obtain consistent results across different plant samples. Additionally, seasonal variations can cause fluctuations in metabolite composition, as certain compounds may be produced at higher levels during specific times of the year. Environmental stressors such as drought, temperature extremes, or pest infestations can also affect the plant’s metabolite production, leading to altered chemical profiles. Furthermore, factors like plant fertility or nutrient status play a crucial role in metabolite accumulation [27], and even allocations of compounds across different plant components (such as roots, leaves, and flowers) can lead to significant variations in extraction results. Understanding these biological and environmental variables is crucial to controlling risks and achieving reliable and reproducible results in medicinal plant extraction. The most common failure modes identified in the medicinal plant extraction process are summarized in Table 1.

**Table 1:** Most Common failure in medical plant extraction [28].

FM	Categories		Error name in the medical plant extraction process	Ref
A1	Sample-related	1	Insufficient sample amount or quality	[29]
A2		2	Inappropriate sample preparation techniques	[30,31]
A3		3	Contamination during sample collection or handling	
A4		4	Volatile material or unstable material affecting metabolite extraction or stability	
A5		5	The freshness of the plant material affects metabolite yield and quality	
A6		6	Selecting appropriate plant tissues for sampling, such as roots, leaves, or flowers, is imperative for ensuring the validity & reliability of research findings	
A7	Extraction method	1	Inadequate extraction solvent or concentration	[32,33]
A8		2	Suboptimal extraction conditions (time, temperature, pH, etc.)	
A9		3	Inefficient extraction technique (solid-liquid extraction, liquid-liquid extraction)	[34]
A10		4	Incompatibility between the extraction method and target metabolites	
A11		5	Inadequate choice of solvent	
A12	Instrumentation, Calibration, and Reference Standards	1	Inaccurate or malfunctioning equipment	[35]
A13		2	Poor calibration or incorrect settings	[36]
A14		3	Contamination in the analytical system	[37]
A15		4	Instrumental and device errors affecting the accuracy and precision of measurements	
A16	Analytical method-related issues	1	Inappropriate choice of analytical technique (chromatography, spectroscopy)	
A17		2	Lack of specificity or sensitivity in the method	[38]
A18		3	Inadequate separation or detection of target metabolites	
A19		4	Matrix interference affecting the analysis	[39]
A20		5	Lack of reference standards for metabolite identification and quantification	[37]
A21	Operator-related issues and data analysis	1	Lack of training or expertise in extraction and analysis techniques	[40]
A22		2	Inconsistent or improper execution of the extraction method	
A23		3	Human error during sample handling analysis, or data processing	
A24		4	Inadequate statistical analysis or data interpretation	
A25	Biological and environmental factors	1	Variability in metabolite content due to plant species, genotype, or developmental stage	[41] [26]
A26		2	Presence of interfering compounds or endogenous substances	[42]
A27		3	Seasonal variations affecting metabolite composition	
A28		4	Impact of environmental stressors on metabolite extraction	
A29		5	Plant fertility or nutrient status influencing metabolite production & accumulation	
A30		6	Allocations of diverse compounds across distinct plant components (roots, leaves, flowers)	

### 1.2. The Research Problem and Research Gaps

The process of medicinal plant extraction plays a crucial role in obtaining bioactive compounds, yet it is fraught with risks that can impact the quality and efficacy of the final product. These risks stem from multiple factors such as sample variability, extraction method selection, instrumentation, and human error. A comprehensive risk analysis is essential to ensure consistency and quality in the extraction process, yet such analyses are often lacking or underdeveloped in current research. Based on the existing literature, it is evident that there is a significant gap in studies investigating the risks in medicinal plant extraction. Furthermore, most studies employing FMEA tend to focus on calculating the Risk Priority Class (RPC) factor, often in controlled environments. However, none of the studies account for the reliability and uncertainty of expert opinions, which are critical for a comprehensive risk framework. This gap highlights the need for a more thorough approach to FMEA, incorporating expert reliability and uncertainty to ensure a more robust and comprehensive risk analysis in medicinal plant extraction.

### 1.3. Research aim and objectives

The aim of this study is to develop and apply an uncertainty-aware risk assessment framework for medicinal plant extraction by integrating FMEA with ZE-number-based decision-making tools, where ZE-BWM is used to weight the risk criteria and (ZE-)MABAC is employed to prioritize failure modes.

- To identify and categorize the main failure modes and risk factors in medicinal plant extraction based on the FMEA structure.
- To collect expert evaluations under uncertainty and reliability considerations using Z-/ZE-number representations and convert them into triangular fuzzy numbers.
- To determine the relative importance (weights) of the FMEA risk criteria using the ZE-BWM method.
- To rank and prioritize the identified failure modes using the (Z/ZE) MABAC approach and provide practical mitigation implications for improving extraction quality and reliability.

### 1.4. Research Novelty and Contributions

Despite the extensive application of FMEA in pharmaceutical and manufacturing processes, its use in medicinal plant extraction remains limited, particularly under conditions of uncertainty and expert judgment reliability. To address these limitations, this study offers the following key contributions:

1. Development of a novel integrated risk assessment framework that combines FMEA with ZE-number-based multi-criteria decision-making methods, enabling a more comprehensive and uncertainty-aware evaluation of risks in medicinal plant extraction processes.
2. Incorporation of expert reliability and consensus into risk analysis by employing ZE-numbers, which extend traditional Z-numbers and overcome the limitations of conventional FMEA approaches that assume equal credibility of expert opinions.
3. Application of the ZE-BWM to objectively determine the relative importance of FMEA risk criteria (Severity, Occurrence, and Detection), reducing inconsistency and subjectivity in criteria weighting.
4. Utilization of the ZE-MABAC method for robust risk prioritization, allowing for a clear ranking of failure modes based on their distance from the border approximation area under fuzzy and uncertain conditions.
5. Provision of practical insights and decision-support guidelines for improving quality control, standardization, and efficiency in medicinal plant extraction, offering a transferable framework applicable to pharmaceutical and herbal medicine industries.

The remainder of this paper is organized as follows. Section 2 reviews Critical Barriers in Medical Plant Extraction identification. Section 3 presents the proposed methodology, including the integration of FMEA with ZE-numbers, the ZE-BWM for criteria weighting, and the ZE-MABAC method for risk prioritization. Section 4 describes the medicinal plant extraction case study and reports the evaluation results. Section 5 discusses the findings and their practical implications for improving extraction quality and process reliability. Finally, Section 6 concludes the paper and outlines directions for future research.

## 2. Research Methodology

**Fig 1:**
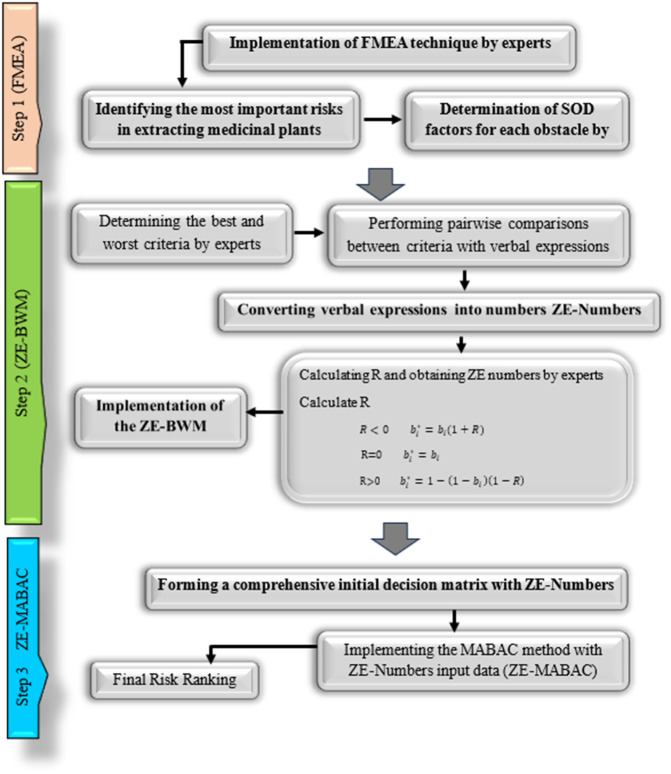
Flowchart of Study.

The flowchart in this study outlines a structured risk assessment process using the integrated FMEA–Z-MABAC method within the ZE-numbers environment (See Fig 1). The process follows eleven key steps. Step 1: Identification of Risk Factors involves gathering expert insights to recognize critical failure modes in areas such as sample handling, extraction conditions, and instrumentation. Step 2: Evaluation Using FMEA Parameters requires experts to assess each risk based on S, O and D using linguistic terms. Step 3: Conversion to ZE-Numbers transforms these linguistic inputs into ZE-numbers, incorporating both fuzziness and expert reliability. Step 4: Construction of the Initial Decision Matrix compiles all ZE-number evaluations into a structured matrix format. Step 5: Normalization of the Decision Matrix ensures that all data are scaled uniformly for comparability across criteria. Step 6: Weight Calculation Using ZE-BWM applies the Best-Worst Method within a ZE-number framework to derive the importance of each criterion. Step 7: Formation of the Weighted Decision Matrix involves multiplying normalized values by the derived weights to reflect the influence of each criterion. Step 8: Calculation of the Border Approximation Area (BAA) establishes reference values that represent the ideal boundary for decision-making. Step 9: Computation of the Substitution Distance Matrix (Q) measures the deviation of each alternative from the BAA. Step 10: Aggregation and Defuzzification of Utility Scores: combine the deviation values and convert them into crisp numerical scores. Finally, Step 11: Ranking of Alternatives ranks the risk factors based on their defuzzified scores, prioritizing those with the greatest impact on medicinal plant extraction. This clear, step-by-step framework ensures a transparent, data-driven, and uncertainty-aware approach to risk prioritization.

### 2.1. Theoretical background of fuzzy and ZE-number-based modeling

#### 2.1.1. Fuzzy membership functions and arithmetic operations.

Fuzzy set theory is employed to model uncertainty in expert evaluations by allowing elements to belong to a set with varying degrees of membership. In this study, triangular fuzzy numbers are used to represent linguistic assessments due to their simplicity and computational efficiency. Basic arithmetic operations on triangular fuzzy numbers are applied to aggregate and transform expert judgments throughout the decision-making process.

#### 2.1.2. Z-numbers.

A Z-number is defined as an ordered pair Z = (A, B), where A represents a fuzzy restriction on a variable, and B denotes the reliability or confidence associated with that restriction. In practical applications, the reliability component B is transformed into a numerical value using a weighting parameter α. The parameter α controls the influence of reliability on the decision-making process by adjusting the contribution of expert confidence relative to the uncertainty captured in A. Therefore, α plays a critical role in balancing uncertainty and reliability in Z-number-based modeling.

#### 2.1.3. ZE-numbers.

ZE-numbers extend the concept of Z-numbers by incorporating group consensus into the reliability assessment. In ZE-numbers, the parameter M represents the consensus matrix derived from expert voting outcomes, including agreement, disagreement, and neutrality. This matrix quantifies the collective evaluation of reliability and enables the adjustment of individual reliability assessments based on group-level consensus. Consequently, the parameter M ensures that the final decision-making process reflects not only individual expert confidence but also the overall agreement within the expert group.

#### 2.1.4. Fuzzy Number Theory.

Zadeh’s [43] pioneering research introduced fuzzy set theory, providing a powerful framework for handling uncertainty and subjectivity across various domains. In fuzzy set theory, a fuzzy set is defined with membership values in the range [0,1]. Consider a fuzzy set U composed of numerical values, where the membership function is represented as: [44]


m: U→[0,1 (1)

and is denoted by μ̃ s (x).

Fuzzy number theory is a branch of mathematics that deals with fuzzy numbers, which are a generalization of classical numbers used to represent uncertain, imprecise, or vague data. Unlike crisp numbers that have a single, precise value, fuzzy numbers are characterized by a range of possible values and a membership function that assigns a degree of membership to each value within this range. Key Concepts in Fuzzy Number Theory:

1. Fuzzy Numbers: These are special types of fuzzy sets defined on the real number line, where each element is associated with a membership value between 0 and 1.
2. Membership Function: This function represents the degree of truth or compatibility of each number within the fuzzy set. Commonly used membership functions include triangular, trapezoidal, and Gaussian shapes.
3. Arithmetic Operations: Fuzzy numbers can undergo operations such as addition, subtraction, multiplication, and division, often defined to preserve their fuzzy nature. Assuming that two TFNs are defined as B̃ = (l₂, m₂, u₂), Ã = (l₁, m₁, u₁) and λ are a constant number and greater than zero, arithmetic operations on these two sets can be defined as Eqs: [45]

$$\tilde{\text{A}}\;⊕\;\tilde{\text{B}}\;=\;({\text{l}}_{\text{1}}+\;{\text{l}}_{\text{2}},\;{\text{m}}_{\text{1}}\;+\;{\text{m}}_{\text{2}},\;{\text{u}}_{\text{1}}\;+\;{\text{u}}_{\text{2}})$$ (2)

$$\tilde{\text{A}}\;⊗\;\tilde{\text{B}}\;=\;({\text{l}}_{\text{1}},\;{\text{l}}_{\text{2}},\;{\text{m}}_{\text{1}},{\text{m}}_{\text{2}},\;{\text{u}}_{\text{1}},\;{\text{u}}_{\text{2}})$$

$$\tilde{\text{A}}\;⊖\;\tilde{\text{B}}\;=\;({\text{l}}_{\text{1}}-{\text{u}}_{\text{2}},{\text{m}}_{\text{1}}\;-\;{\text{m}}_{\text{2}},\;{\text{u}}_{\text{1}}\;-\;{\text{l}}_{\text{2}})$$

$$\tilde{\text{A}}⊘\tilde{\text{B}}\;=\;({\text{l}}_{\text{1}}/\;{\text{u}}_{\text{2}},\;{\text{m}}_{\text{1}}/\;{\text{m}}_{\text{2}},\;{\text{u}}_{\text{1}}/\;{\text{l}}_{\text{2}})$$

$$\lambda \;\tilde{\text{A}}\;=\;(\lambda \;{\text{l}}_{\text{1}},\;\lambda {\text{m}}_{\text{1}},\;\lambda {\text{u}}_{\text{1}})$$

1. In fuzzy numbers, the definitive number R(ã) can be calculated for the triangular fuzzy set (l, m, u) using R(ã) = (l + 4m + u)/6 indicating the concept of graded mean integration representation (GMIR). [45]
2. Applications: Fuzzy number theory is widely used in decision-making, optimization, control systems, and areas where data is incomplete, uncertain, or imprecise [46]

By providing a flexible way to handle uncertainty, fuzzy number theory plays a crucial role in fields such as artificial intelligence, economics, engineering, and risk analysis. Identified barriers and risks in the extraction process of medicinal plants.

#### 2.1.5. Triangular Fuzzy Numbers (TFNs).

A significant category of fuzzy numbers is triangular fuzzy numbers (TFNs), which emphasize the hierarchical structure of membership values. A TFN is represented by three components (l, m, u), which provide insights into:

L is Lower value; m: Central value and u: Upper value. The mathematical formulation and functional properties of TFNs are expressed in the following equation: [47]


TFN=(l,m,u) (3)

$$\begin{array}{c} {\tilde{\mu }}_{s}(x)=\left\{ \begin{array}{c} @c\text{0}\;,\;x<l\\ \frac{x-l}{m-l},\;l\leq x\leq m\\ \frac{u-x}{u-m},\;m\leq x\leq u\\ \text{0},\;x>u \end{array}\right.\; \end{array}$$ (4)

A Z-number consists of a pair of fuzzy numbers, represented as Z = (A, B) [43]. In this representation, component A denotes the fuzzy constraint variable within domain X, while component B evaluates the reliability of A using TFNs. The probabilistic constraint, denoted as R(X), defines the potential distribution [44]. This constraint can be formulated using the following equation [48]


$$R(X):X\;is\;A\;\to Poss\;(X=u)=u_{A}\;(u)$$ (5)

In Equation 2, u represents the general value of X, while $u_{A}$ serves as the membership function for A, acting as a constraint related to R(X). The probability distribution representation of R(X) is explained in Equation 6 [48]


R(X):X is P →Prob (u≤x≤u+du)=p(u)du (6)

Using Equation 7, the reliability aspect of Z-numbers is converted into a crisp value


$$\propto =\frac{\int x\mu_{B}dx}{\int \mu_{B}dx}$$ (7)

According to the fundamental concept introduced by Zadeh (2011) [43], the structure of Z-numbers incorporates an inherent probabilistic relationship between components A and B, as expressed in the following equation.


$$\sum_{i=\text{1}}^{n}\mu_{A}(x_{i}).\;p_{x_{A}}\left(x_{i}\right)\to \;b_{i}$$ (8)

#### 2.1.6. Fuzzy ZE-Numbers.

A Z-number is defined as an ordered pair *Z* = (A, B), where A represents a fuzzy restriction on the values of a variable, and B expresses the reliability or certainty associated with this restriction. While Z-numbers effectively model uncertainty by combining fuzzy information with reliability, they assume that expert judgments are provided individually and do not explicitly account for group consensus. To ensure maximum objectivity, the data collected from Z-number decision-makers should strive for complete neutrality. In the study by Tian et al. [33], an advancement called ZE-numbers was introduced, aiming to enhance the evaluation of group decision-making reliability beyond the capabilities of Z-numbers.

ZE-numbers extend the concept of Z-numbers by incorporating an additional evaluation-counting component that reflects the level of agreement among experts in a group decision-making environment. In ZE-numbers, the reliability component is adjusted based on the number of experts who agree, disagree, or remain neutral regarding a given assessment. As a result, ZE-numbers provide a more realistic and robust representation of uncertainty by simultaneously considering fuzziness, reliability, and group consensus. In this study, ZE-numbers represented by triangular fuzzy numbers (referred to as Fuzzy ZE-numbers) are employed to enhance the credibility of expert-based risk assessments in medicinal plant extraction. The structure of ZE-numbers is formulated as shown in Equation 9 [49]


 ZE=((A,B),E) (9)

In the evaluation of group decision-making reliability, the approach adopted by Tian et al. [49] involved the use of a voting method. Where three decision-makers first provide their assessments in the form of Z-numbers by assigning fuzzy membership values and reliability levels. Subsequently, a group of experts evaluates these Z-number assessments through a voting mechanism (agree/disagree/neutral), represented by *Y*, *N* and *θ*, to construct ZE-numbers that reflect group consensus. As shown in Equation 10, in this group decision-making voting technique, Y represents the number of experts who agree with the assessed Z-numbers, N denotes the number of experts who disagree, and θ accounts for the number of neutral experts.


Evaluation−number=(Y, N, θ) (10)

The component denoted as E in Equation 8 represents evaluation-counting, which reflects individual assessments through group consensus to establish decision credibility. This element E encompasses the inherent reliability of both elements A and B. When converting a Z-number into a ZE-number, the use of Equations 11–12 provides a feasible solution [48]


$$M=\left\{ \begin{array}{c} \;\;\;b_{i}^{*}=b_{i}(\text{1}+R)\;\;\;\;\;.\;R<\text{0}\\ \;\;\;b_{i}^{*}=b_{i}.\;\;\;\;\;\;\;\;.\;R=\text{0}\\ \;\;\;b_{i}^{*}=\text{1}-\left(\text{1}-b_{i}\right)(\text{1}-R)\;.\;R>\text{0} \end{array}\right.\;$$ (11)

$$R=\frac{Y-N}{n-\theta }$$ (12)

Here, the notation $b_{i}^{*}$ specifically represents the adjusted or refined value assigned to b_i_. In this context, b_i_ stands for the intrinsic b value in component B of Z-numbers. Meanwhile, the variable n is significant as it quantifies the total number of participants involved in the process [49].

### 2.2. FMEA theory

FMEA is one of the most important and widely used methods for risk assessment and prioritization due to its simplicity and low cost [50] Large organizations, such as NASA, have employed this method for years in risk management, evaluation, identification, and prioritization [50]. This approach allows experts to rank specified criteria from highest to lowest importance, aiding organizations in developing proactive planning. To enhance accuracy, MCDM methods can be utilized [51,52]. In this study, the Z-MABAC and ZE-MABAC methods are applied to rank risks and failure modes. FMEA is a critical systematic tool employed in various industries to analyze potential failure modes and their consequences [53]. By dissecting complex systems into manageable subsystems, FMEA enables teams to identify possible failures associated with each component or function [53]. This proactive approach is particularly valuable during the early stages of product and process development, as it allows organizations to detect vulnerabilities and implement preventive measures before they escalate into significant issues [53]. The structured nature of FMEA fosters a thorough examination of products and processes, ensuring that potential failures are not overlooked [9].

One of the key advantages of FMEA is its ability to enhance the design and development of new products, processes, or services [50]. By documenting parameters and indicators throughout the design and development phases, teams can create a robust framework that supports the selection of high-reliability solutions. This meticulous documentation also aids in establishing criteria for planning necessary tests and setting reliability benchmarks, which are essential for achieving long-term success. Furthermore, FMEA encourages teams to identify and prioritize corrective actions, ultimately reducing the severity of failure effects through strategic measures [54]. To maximize the effectiveness of FMEA, it is crucial to involve expert groups with specialized knowledge and experience. Their insights significantly increase the accuracy of the analysis and the reliability of outcomes. The simplicity and ease of implementation of FMEA make it an attractive option for organizations aiming to manage risks effectively. By adopting a holistic approach to risk reduction, FMEA not only enhances the ability to detect failures before products reach customers but also contributes to the overall documentation of the design and manufacturing process. This comprehensive methodology ultimately leads to improved product reliability and customer satisfaction [55].

In the FMEA method, priority values confirmed by experts are used to rank failure modes and errors based on three key indices: Severity (S), Occurrence (O), and Detection (D) [56]. Each risk factor is rated on a numerical scale from 1 (least probable, least severe, most detectable) to 10 (most probable, most severe, least detectable). The highest possible RPN is 1000, and the lowest is 1. A higher RPN indicates a higher priority for risk mitigation. Once risks are identified, corrective actions are implemented. Failure modes are then re-evaluated to determine whether corrective actions have effectively reduced risks. The RPN is calculated by multiplying the three indices using the following formula: [56,57]


RPN=S×O×D (13)

Corrective actions are considered effective in reducing risks and their effects if the recalculated RPN decreases. It is important to note that corrective actions are implemented starting from the highest RPN values. As observed in the RPN formula, all indices (S, O, and D) are treated with equal weight, meaning that the importance of one factor over another is not considered [52]To address this issue, decision-making approaches such as Analytical Hierarchy Process (AHP), Data Envelopment Analysis (DEA), Saw method, and other multi-criteria decision-making (MCDM) techniques can be applied [52].

### 2.3. Best-Worst Method (BWM)

The BWM was first introduced by Rezaei in 2015 [58]. This method is considered one of the most recent techniques for weighting indicators and criteria in decision-making problems [59]. One of the key features of BWM is its ability to reduce the number of pairwise comparisons between decision-making criteria compared to methods such as AHP (Analytic Hierarchy Process). In BWM, decision makers first rank all criteria from the most important to the least important and then compare each criterion pairwise. However, in BWM, decision makers initially select the best and worst criteria and, in the second step, compare the remaining criteria with these two reference points [59]. This significantly reduces time and cost. The method initially formulates a nonlinear optimization problem, which can be easily converted into a linear form. Solving this problem yields the weights of the criteria, where the absolute differences in weights are observed to be quite small. To ensure the reliability of the obtained results, since they are based on decision makers opinions, the consistency ratio is used [59]. As previously mentioned, BWM benefits from fewer comparisons, more robust assessments, and higher confidence in results, making it a suitable choice for this study. Since decision makers opinions are qualitative and based on their personal experiences, they inherently contain uncertainty, vagueness, and ambiguity in real-world applications [60]. In traditional BWM, decision makers conduct their comparisons using crisp natural numbers between 1 and 9, which limits the method’s ability to account for uncertainty and imprecision in decision makers’ judgments [60]. To address this issue, Zhao et al. [47] extended BWM into a fuzzy environment, incorporating uncertainty into the calculations. Their study demonstrated that using fuzzy sets in decision makers assessments is more efficient than the traditional BWM, leading to a significantly lower inconsistency ratio [60,61]. In this paper, we advance the concept of BWM using ZE-numbers and Z-numbers. By solving this model, the optimal weights are obtained. In this article, we explore the BWM by incorporating fuzzy numbers ZE and Z to enhance decision-making accuracy. The methodology follows a structured approach consisting of the following key steps:

***Step 1: Defining the Criteria and Decision-Makers***

The first step involves identifying the set of criteria relevant to the decision-making process. Additionally, we determine the decision-makers who will provide evaluations based on their expertise. These individuals play a crucial role in assessing the importance of each criterion. In this study, three decision-makers were selected based on their professional experience and technical expertise in medicinal plant extraction, laboratory analysis, and risk assessment. Each decision-maker had several years of hands-on experience in pharmaceutical or herbal processing environments, ensuring informed and reliable evaluations of the FMEA risk criteria. To ensure the consistency and reliability of the decision-makers’ judgments, the BWM consistency ratio (CR) was calculated for each set of pairwise comparisons. The obtained consistency ratios were all within the acceptable threshold, confirming that the judgments provided by the decision-makers are logically consistent and suitable for subsequent weighting and ranking analyses.

***Step 2: Pairwise Comparisons Using BO and OW***

In this step, decision-makers compare the best criterion with the other criteria, a process referred to as Best-to-Others (BO) comparison. Similarly, they compare all other criteria with the worst criterion, known as Others-to-Worst (OW) comparison. These comparisons are performed using linguistic terms, which are then converted into TFN for precise quantification.

***Step 3: Developing a Nonlinear Programming Model***

After obtaining the pairwise comparisons, we formulate a nonlinear programming model to derive the optimal weights of the criteria. This model ensures consistency in the decision makers’ judgments and provides a reliable ranking of the criteria based on their relative importance. By solving this optimization model, we obtain the final fuzzy weights, which can be used for more informed and accurate decision-making in complex multi-criteria problems. This approach enhances the traditional BWM by integrating fuzzy logic, thereby addressing uncertainties in decision makers evaluations [47].


$$\begin{array}{cc} & min\xi^{k}\\ & \left\{ \begin{array}{c} \left|\frac{\left(l_{B}^{w},m_{B}^{w},u_{B}^{w}\right)}{\left(l_{j}^{w},m_{j}^{w},u_{j}^{w}\right)}-\left(l_{Bj},m_{Bj},u_{Bj}\right)\right|\leq \left(k^{*},k^{*},k^{*}\right)\mathrm{\backslash vspace}1.5mm\\ \;\;\left|\frac{\left(l_{j}^{w},m_{j}^{w},u_{j}^{w}\right)}{\left(l_{W}^{w},m_{W}^{w},u_{W}^{w}\right)}-\left(l_{jW},m_{jW},u_{jW}\right)\right|\leq \left(k^{*},k^{*},k^{*}\right)\\ \;\;\;\;\;\;\;\sum {\mathrm{\backslash nolimits}}_{j=\text{1}}^{n}\;R\left({\tilde{w}}_{j}\right)=\text{1}\\ \;\;\;\;\;\;\sum {\mathrm{\backslash nolimits}}_{j=\text{1}}^{w}\;\leq m_{j}^{w}\leq u_{j}^{w}\\ \;\;\;\;\;\;\;\;\;\;l_{j}^{w}\geq \text{0}\\ \;\;\;\;\;\;\;\;j=\text{1},\text{2},\ldots ,n \end{array}\right.\; \end{array}$$ (14)

In this study, decision makers judgments are first expressed as Z-numbers or ZE-numbers and subsequently transformed into triangular fuzzy numbers (l, m, u). These TFNs are then employed as inputs to the fuzzy BWM model for criteria weighting.

### 2.4. Linguistic variables of membership functions for decision makers judgment

The table presents linguistic terms used in decision makers judgment and decision-making, particularly in fuzzy logic or MCDM methods. It categorizes different levels of importance into six linguistic terms: Equally Important (EI), Weakly Important (WI), Fairly Important (FI), Important (I), Very Important (VI), and Absolutely Important (AI). Each term is associated with a membership function, represented as a fuzzy number triplet, which helps quantify qualitative importance in a decision-making process. The table also provides Consistency Index Scores (CIs) for each linguistic term, which increase from 3.00 for Equally Important to 9.35 for Absolutely Important, reflecting the relative weight of each term. These fuzzy numbers and CIs are essential in handling uncertainty in decision makers evaluations and are often used in fuzzy AHP (Analytic Hierarchy Process) or other fuzzy decision-making techniques to ensure systematic and consistent judgments. The linguistic terms and triangular fuzzy numbers used for decision-makers’ judgments and reliability assessments are presented in Tables 2 and 3, respectively.

**Table 2:** Linguistic Variables [34].

Linguistics terms	Membership function	CIs
Equally Important (EI)	(1,1,1)	3.00
Weakly important (WI)	(2/3,1, 3/2)	3.8
Fairly important (FI)	(3/2,2,5/2)	5.29
Important (I)	(5/2,3,7/2)	6.69
Very important (VI)	(7/2,4,9/2)	8.04
Absolutely important (AI)	(9/2,5,11/2)	9.35

**Table 3:** Linguistic variables of reliablities.

Linguistic variables	Very High(VH)	High(H)	Medium(M)	Low(L)	Very Low (VL)
TFNs	(0.7,1.0,1.0)	(0.5,0.7,0.9)	(0.3,0.5,0.7)	(0.1,0.3,0.5)	(0,0,0.3)

Tables 2 and 3 jointly define the two components of Z-numbers, where Table 2 represents the fuzzy restriction (uncertainty) and Table 3 provides the corresponding reliability (confidence) values.

#### 2.4.1. Pairwise comparisons of each decision-maker.

In this table, linguistic variables have been converted into TFN. To validate the obtained results, the Consistency Ratio (CR) must be calculated. For this purpose, the following formula is used, where $\;{\tilde{\xi }}^{*}$ a higher value results in a larger CR, indicating lower reliability of the pairwise comparisons. The CR is considered acceptable when its value is less than 0.1 [58]


$$CR=\frac{{\tilde{\xi }}^{*}}{CI}$$ (15)

The table represents a decision-making process where multiple decision-makers (TM1, TM2, and TM3) evaluate three criteria Severity (S), Occurrence (O), and Detection (D)—using linguistic variables. The evaluation is divided into two sections: the BO (Best Option) vector of risk factors and the OW (Out Worst) vector of risk factors. In the BO section, each decision-maker selects the best criterion among the three and then compares it with the others using linguistic terms such as Equally Important (EI), Weakly Important (WI), Fairly Important (FI), and Very Important (VI). For example, TM1 has chosen Detection (D) as the best criterion and compared it with Severity (S) and Occurrence (O), assigning “Important (I)” for D vs. S and “Weakly Important (WI)” for D vs. O. Similarly, in the OW section, each decision-maker selects the worst criterion and evaluates the other two in relation to it. TM1, for instance, has identified Occurrence (O) as the worst criterion and compared it with S and D, using “Important (I)” for S vs. O and “Fairly Important (FI)” for D vs. O. This approach helps in systematically analyzing risk factors from both best and worst perspectives, ensuring a balanced and structured decision makers evaluation. The pairwise comparison results provided by the three-decision maker using linguistic variables are reported in Table 4.

**Table 4:** decision-making by 4 decision makers.

	BO vector of risk factors					OW vector of risk factors				
			S	O	D			S	O	D
	Best					Worst				
TM1	D		I	WI	EI	O		I	EI	FI
TM2	S	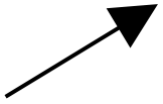	EI	FI	VI	D	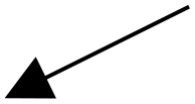	AI	WI	EI
TM3	O		WI	EI	I	S		EI	I	VI

It should be noted that in the BWM framework, the logical validity of the comparisons is assessed through the consistency ratio (CR) derived from the optimization model, rather than by imposing identical or maximum numerical values for specific pairwise comparisons.

#### 2.4.2. Linguistic variables.

The table represents linguistic variables of reliabilities and their corresponding TFNs. Linguistic variables are qualitative descriptors used to express the reliability of a criterion or decision in a fuzzy environment. Each linguistic variable is assigned a TFN, which is a set of three values representing the lower, middle, and upper bounds of the fuzzy number. This table is essential for fuzzy decision-making models, where reliability assessments are expressed in linguistic terms and then converted into numerical values for processing. By using TFNs, uncertainties and subjectivity in decision makers evaluations are better represented, leading to more accurate and flexible decision analysis (see Table 3).

The tables present the fuzzy weights obtained using the BWM, where the reliability linguistic variables in Z and ZE numbers have been converted into Triangular Fuzzy Numbers (TFN). The BWM method has been applied to determine the weights of the criteria using both Z-BWM and ZE-BWM approaches. The nonlinear programming model described in the BWM methodology was solved using LINGO software, and the resulting fuzzy weights are displayed in the tables. The left table corresponds to ZE-BWM fuzzy weights, while the right table represents Z-BWM fuzzy weights for three decision-makers (TM1, TM2, and TM3). Each decision-maker evaluated three criteria: S, O, and D, assigning fuzzy weight values based on their importance. These weights provide a structured and systematic approach for prioritizing risk factors in decision-making scenarios. The fuzzy weights of the FMEA criteria obtained using the ZE-BWM method are shown in Table 4.

### 2.5. Ethics approval and consent to participate

This study involved an expert survey administered as a paper-based questionnaire to support risk assessment for plant-extract methodology. No personal, sensitive, or identifiable information was collected, and all results are reported in aggregated form. Participation was voluntary. Informed consent was obtained prior to participation by providing each expert with an information sheet explaining the study aims, procedures, expected time required, confidentiality and data handling, and the right to decline or withdraw. Participants’ completion documented consent and return of the questionnaire, consistent with minimal-risk survey research practice. No minors were involved. Ethical approval was exempted for this non-interventional study involving non-identifiable expert opinions, according to institutional guidance.

## 3. Evaluation Results of Decision-Makers and Experts Judgments

In the Z-number framework, the reliability component is determined solely based on the judgment of an individual decision-maker. However, ZE-numbers extend this concept by incorporating group consensus through an evaluation-counting mechanism. In this study, three decision-makers first provide their assessments in the form of Z-numbers by assigning fuzzy membership values and corresponding reliability levels. Subsequently, a group of experts evaluates these Z-number assessments using a voting mechanism consisting of agreement, disagreement, or neutrality, denoted by Y, N, and θ, respectively. Based on the voting results, the reliability component of each Z-number is adjusted to reflect the level of consensus among experts, leading to the construction of ZE-numbers. This adjustment enables the proposed framework to account not only for uncertainty and reliability but also for collective agreement, thereby providing a more robust representation of expert knowledge in group decision-making environments. After constructing the ZE-numbers, they are transformed into triangular fuzzy numbers (l,m,u) and subsequently employed as inputs to the ZE-BWM and ZE-MABAC procedures. All remaining computational steps follow the same formulation described in the previous section and are therefore omitted for brevity.

Based on the above explanations, the calculations for generating ZE-numbers have been performed, and the results are presented in the two tables below. Table 5 presents the results of expert evaluations using ZE-numbers, a methodology that extends fuzzy set theory to incorporate uncertainty in decision-making. The table is derived from linguistic assessments provided by experts, who evaluated different risk factors associated with the medicinal plant extraction process. The evaluations were converted into TFNs and further refined using the ZE-number approach, which integrates both expert reliability and consensus through a voting mechanism. The parameters Y (number of agreeing experts), N (number of disagreeing experts), and θ (neutral experts) were used to compute reliability scores (R) for each assessment. These scores reflect the confidence level in each expert judgment, ensuring a more robust and structured risk evaluation framework. Table 6 presents the ZE-number evaluations derived from the expert judgments described.

**Table 5:** fuzzy weights, ZE-BWM Weight.

TM1	
S	(0.241,0.268,0.347)
O	(0.122,0.122,0.14)
D	(0.587,0.587,0.648)
TM2	
S	(0.581,0.581,0.627)
O	(0.212,0.237,0.299)
D	(0.155,0.164,0.19)
TM3	
S	(0.161,0.161,0.193)
O	(0.516,0.516,0.597)
D	(0.26,0.288,0.4)

**Table 6:** ZE-number evaluations of risk factors in medicinal plant extraction, incorporating expert 1, 2 and 3 consensus and reliability assessment.

TM1								TM2							TM 3							
	**S**	**O**	**D**	**Y**	**N**	**θ**	**R**	**S**	**O**	**D**	**Y**	**N**	**θ**	**R**	**S**	**O**	**D**	**Y**	**N**	**θ**		**R**
A1	(M,M)	(MH,VH)	(MH,L)	8	2	2	0.60	(MH,H)	(VH,H)	(VH,M)	9	1	2	0.80	(MH,VH)	(VH,H)	(VH,H)	8	2	2		0.60
A2	(MH, H)	(M, M)	(M,L)	8	3	1	0.45	(H,H)	(MH,H)	(MH,VH)	8	3	1	0.45	(ML,L)	(H,M)	(H,H)	8	3	1		0.45
A3	(MH, L)	(MH, H)	(MH,H)	6	5	1	0.09	(H,L)	(MH,H)	(H,L)	5	5	2	0.00	(M,L)	(MH,H)	(MH,H)	6	5	1		0.09
A4	(M, VH)	(M, M)	(M,M)	1	8	3	−0.78	(ML,M)	(M,L)	(MH,M)	1	8	3	−0.78	(ML,M)	(M,H)	(M,ML)	1	8	3		−0.78
A5	(MH, VH)	(M, M)	(ML,M)	4	4	4	0.00	(L,VL)	(M,M)	(MH,M)	4	4	4	0.00	(ML,M)	(MH,L)	(M,L)	6	4	2		0.20
A6	(MH, M)	(H, H)	(M,M)	3	6	3	−0.33	(MH,H)	(MH,H)	(M,M)	3	6	3	−0.33	(VH,M)	(M,H)	(MH,VH)	3	6	3		−0.33
A7	(MH, L)	(M, H)	(MH,L)	6	4	2	0.20	(ML,M)	(MH,H)	(MH,VH)	6	4	2	0.20	(M,M)	(ML,L)	(M,L)	6	5	1		0.09
A8	(H, VH)	(M, L)	(ML, L)	4	7	1	−0.27	(L,VL)	(M,M)	(MH,H)	4	7	1	−0.27	(MH,M)	(H,H)	(ML,H)	4	7	1		−0.27
A9	(L, M)	(VH, VH)	(L, VL)	9	1	2	0.80	(VH,H)	(VH,H)	(MH,L)	8	1	3	0.78	(H,H)	(ML,M)	(VH,M)	7	3	2		0.40
A10	(H,VH)	(MH,M)	(ML,M)	6	4	2	0.20	(ML,H)	(MH,L)	(VH,H)	6	4	2	0.20	(MH,VH)	(H,M)	(MH,M)	6	4	2		0.20
A11	(M,M)	(MH,VH)	(L,M)	3	8	1	−0.45	(M,M)	(M,L)	(M,VL)	3	7	2	−0.40	(ML,M)	(MH,H)	(M,VL)	3	8	1		−0.45
A12	(ML,L)	(M,VH)	(VL,M)	7	2	3	0.56	(L,L)	(M,L)	(ML,L)	7	2	3	0.56	(VL,VL)	(L,VH)	(M,M)	7	2	3		0.56
A13	(M,L)	(VH,M)	(VL,L)	5	5	2	0.00	(MH,H)	(H,M)	(ML,H)	5	5	2	0.00	(M,L)	(H,M)	(VH,VH)	5	5	2		0.00
A14	(M,L)	(ML,VL)	(ML,H)	2	10	0	−0.67	(ML,M)	(MH,L)	(L,L)	3	8	1	−0.45	(ML,M)	(M,L)	(H,H)	4	7	1		−0.27
A15	(MH,H)	(MH,M)	(ML,VH)	4	6	2	−0.20	(M,M)	(H,M)	(VH,H)	4	6	2	−0.20	(M,H)	(MH,H)	(MH,L)	4	6	2		−0.20
A16	(ML,L)	(M,VH)	(ML,VL)	4	4	4	0.00	(ML,M)	(L,VL)	(H,M)	4	4	4	0.00	(M,L)	(ML,L)	(M,VH)	4	4	4		0.00
A17	(MH,L)	(H,M)	(MH,H)	6	2	4	0.50	(H,H)	(H,H)	(VH,H)	6	2	4	0.50	(MH,VH)	(H,H)	(VH,M)	6	2	4		0.50
A18	(H,VH)	(MH,M)	(VH,H)	4	5	3	−0.11	(MH,VH)	(H,H)	(MH,VH)	3	6	3	−0.33	(MH,L)	(MH,VH)	(H,H)	4	5	3		−0.11
A19	(M,M)	(ML,VL)	(ML,VH)	4	3	5	0.14	(MH,M)	(M,M)	(MH,L)	4	3	5	0.14	(ML,L)	(ML,VL)	(MH,L)	3	4	5		−0.14
A20	(ML,M)	(MH,H)	(ML,VH)	5	3	4	0.25	(ML,M)	(ML,M)	(ML,M)	5	3	4	0.25	(L,L)	(L,MH)	(M,M)	5	3	4		0.25
A21	(ML,VH)	(ML,VH)	(L,L)	5	6	1	−0.09	(L,VL)	(MH,M)	(ML,M)	5	6	1	−0.09	(ML,M)	(L,VH)	(MH,L)	5	6	1		−0.09
A22	(M,M)	(MH,H)	(M,L)	3	8	1	−0.45	(MH,M)	(VH,H)	(VH,L)	2	9	1	−0.64	(H,H)	(MH,M)	(VH,M)	3	8	1		−0.45
A23	(L,L)	(MH,M)	(VL,H)	9	2	1	0.64	(ML,VL)	(M,M)	(ML,L)	9	2	1	0.64	(VL,VL)	(VL,VH)	(MH,H)	7	4	1		0.27
A24	(M,VH)	(M,M)	(ML,M)	2	8	2	−0.60	(ML,M)	(M,L)	(M,M)	2	8	2	−0.60	(ML,VL)	(ML,VH)	(M,L)	2	8	2		−0.60
A25	(MH,VH)	(MH,H)	(M,H)	3	7	2	−0.40	(MH,H)	(M,VH)	(MH,L)	1	7	4	−0.75	(M,H)	(M,VH)	(MH,H)	5	5	2		0.00
A26	(L,VH)	(ML,L)	(L,VL)	7	4	1	0.27	(VL,VL)	(MH,H)	(VH,H)	7	4	1	0.27	(M,VL)	(L,VH)	(MH,VH)	7	4	1		0.27
A27	(L,L)	(M,H)	(VL,VH)	4	3	5	0.14	(M,M)	(ML,L)	(ML,M)	4	3	5	0.14	(ML,M)	(L,VL)	(M,VL)	4	3	5		0.14
A28	(M,H)	(L,VL)	(L,L)	2	10	0	−0.67	(MH,VH)	(ML,VL)	(ML,VL)	4	1	7	0.60	(M,VH)	(ML,VH)	(VH,M)	2	7	3		−0.56
A29	(MH,H)	(M,H)	(ML,VH)	7	3	2	0.40	(MH,H)	(VH,H)	(MH,M)	7	3	2	0.40	(M,H)	(MH,VH)	(MH,M)	7	3	2		0.40
A30	(MH,M)	(M,M)	(VL,M)	4	5	3	−0.11	(ML,M)	(ML,M)	(ML,H)	5	4	1	−0.11	(MH,M)	(ML,L)	(M,VH)	4	5	3		−0.11

In Table 6, the expert voting results (Y, N, and θ) reflect agreement with the overall Z-number evaluation obtained from the combined severity (S), occurrence (O), and detection (D) assessments for each failure mode, rather than with each factor individually.

Table 7 presents the calculated ZE-numbers derived from expert evaluations, structured into a decision matrix format. These values are generated based on the fuzzy assessments of risk factors in the medicinal plant extraction process. The table quantifies expert opinions using TFNs and applies the ZE-number methodology to integrate uncertainty and reliability in decision-making. By converting linguistic variables into numerical values, this table provides a structured representation of risk priorities, facilitating more accurate and data-driven risk analysis. The final decision matrix constructed based on ZE-number evaluations is reported in Table 7.

**Table 7:** Decision matrix with computed ZE-numbers for evaluating risk factors in medicinal plant extraction from the input data collected by decision-makers and experts.

TM1										TM 2									TM3								
	S			O			D			S			O			D			S			O			D		
A1	3.59	4.49	5.39	4.96	6.45	7.93	4.30	5.59	6.88	4.86	6.32	7.78	7.78	8.76	9.73	7.60	8.55	9.50	4.96	6.45	7.93	7.56	8.51	9.45	7.56	8.51	9.45
A2	4.62	6.01	7.40	3.43	4.29	5.15	3.21	4.02	4.82	6.47	7.40	8.32	4.62	6.01	7.40	4.94	6.43	7.91	1.61	2.81	4.02	6.01	6.86	7.72	6.47	7.40	8.32
A3	3.20	4.16	5.12	4.35	5.66	6.96	4.35	5.66	6.96	4.14	4.73	5.32	4.28	5.57	6.85	4.14	4.73	5.32	2.56	3.20	3.84	4.35	5.66	6.96	4.90	6.38	7.85
A4	1.85	2.31	2.77	1.36	1.69	2.03	1.36	1.69	2.03	0.68	1.19	1.69	1.12	1.39	1.67	1.69	2.20	2.71	0.68	1.19	1.69	1.61	2.02	2.42	1.36	1.69	2.03
A5	4.89	6.36	7.83	2.88	3.59	4.31	1.44	2.52	3.59	0.20	0.41	0.61	2.88	3.59	4.31	2.88	3.59	4.31	1.57	2.74	3.92	3.46	4.50	5.54	2.77	3.46	4.16
A6	2.93	3.81	4.70	4.89	5.59	6.29	2.35	2.93	3.52	3.50	4.54	5.59	3.50	4.54	5.59	2.35	2.93	3.52	4.70	5.28	5.87	2.80	3.50	4.20	4.00	5.20	6.39
A7	3.46	4.50	5.54	3.55	4.43	5.32	3.46	4.50	5.54	1.57	2.74	3.92	4.43	5.77	7.10	4.92	6.39	7.87	2.99	3.74	4.49	1.28	2.24	3.20	1.28	2.24	3.20
A8	5.84	6.68	7.51	2.02	2.52	3.03	0.50	1.01	1.51	0.17	0.35	0.52	2.45	3.06	3.68	3.65	4.75	5.84	3.06	3.98	4.90	5.11	5.84	6.57	1.46	2.56	3.65
A9	0.95	1.90	2.85	7.97	8.96	9.96	0.97	1.95	2.92	7.76	8.73	9.70	7.76	8.73	9.70	4.62	6.01	7.40	6.42	7.33	8.25	1.69	2.95	4.21	5.90	6.74	7.58
A10	6.88	7.87	8.85	3.92	5.09	6.27	1.57	2.74	3.92	1.77	3.10	4.43	4.85	5.54	6.24	7.10	7.98	8.87	4.92	6.39	7.87	5.48	6.27	7.05	3.92	5.09	6.27
A11	2.12	2.65	3.19	3.61	4.70	5.78	0.53	1.06	1.59	2.23	2.78	3.34	1.83	2.29	2.75	0.63	0.79	0.95	1.06	1.86	2.65	3.16	4.11	5.06	0.60	0.75	0.90
A12	1.69	2.95	4.22	3.96	4.95	5.94	0.00	0.89	1.77	0.84	1.69	2.53	3.37	4.22	5.06	1.69	2.95	4.22	0.00	0.76	1.52	0.99	1.98	2.97	3.54	4.43	5.32
A13	1.18	2.07	2.96	5.75	6.47	7.19	0.59	1.18	1.77	4.28	5.57	6.85	5.03	5.75	6.47	1.71	3.00	4.28	2.37	2.96	3.55	5.03	5.75	6.47	7.83	8.81	9.79
A14	1.37	1.71	2.05	0.24	0.41	0.59	0.99	1.73	2.47	1.06	1.86	2.65	2.18	2.84	3.50	0.44	0.87	1.31	1.23	2.15	3.06	2.02	2.52	3.03	5.11	5.84	6.57
A15	3.83	4.98	6.13	3.21	4.18	5.14	1.75	3.06	4.38	2.57	3.21	3.86	4.50	5.14	5.79	6.13	6.89	7.66	3.06	3.83	4.60	3.83	4.98	6.13	2.65	3.44	4.23
A16	1.18	2.07	2.96	3.92	4.89	5.87	0.41	0.71	1.02	1.44	2.52	3.59	0.20	0.41	0.61	5.03	5.75	6.47	2.37	2.96	3.55	1.18	2.07	2.96	3.92	4.89	5.87
A17	4.11	5.34	6.57	6.10	6.97	7.84	4.65	6.05	7.45	6.52	7.45	8.38	6.52	7.45	8.38	7.45	8.38	9.31	4.95	6.43	7.92	6.52	7.45	8.38	6.97	7.84	8.71
A18	6.46	7.38	8.31	3.39	4.40	5.42	6.46	7.27	8.07	4.00	5.20	6.39	4.89	5.59	6.29	4.00	5.20	6.39	2.79	3.63	4.46	4.61	6.00	7.38	5.65	6.46	7.27
A19	3.06	3.83	4.59	0.85	1.48	2.11	1.96	3.44	4.91	3.83	4.97	6.12	3.06	3.83	4.59	3.33	4.33	5.32	1.10	1.92	2.74	0.38	0.66	0.94	2.74	3.56	4.38
A20	1.60	2.79	3.99	4.47	5.81	7.16	1.97	3.44	4.92	1.60	2.79	3.99	1.60	2.79	3.99	1.60	2.79	3.99	0.72	1.43	2.15	0.80	1.60	2.40	3.19	3.99	4.79
A21	1.87	3.27	4.67	1.87	3.27	4.67	0.56	1.13	1.69	0.19	0.39	0.58	3.43	4.45	5.48	1.37	2.40	3.43	1.37	2.40	3.43	0.82	1.63	2.45	2.82	3.67	4.51
A22	2.12	2.65	3.19	3.16	4.11	5.06	1.75	2.18	2.62	2.17	2.82	3.47	4.13	4.65	5.16	2.85	3.21	3.57	4.43	5.06	5.69	2.65	3.45	4.25	4.25	4.78	5.31
A23	0.87	1.75	2.62	4.54	5.90	7.26	0.00	0.95	1.90	1.61	2.83	4.04	3.63	4.54	5.45	1.75	3.06	4.37	0.00	0.55	1.10	0.00	0.98	1.97	4.49	5.84	7.18
A24	2.48	3.10	3.71	1.82	2.27	2.73	0.91	1.59	2.27	0.91	1.59	2.27	1.50	1.87	2.24	1.82	2.27	2.73	0.26	0.45	0.65	1.24	2.17	3.10	1.50	1.87	2.24
A25	3.79	4.93	6.07	3.32	4.31	5.31	2.65	3.32	3.98	2.14	2.78	3.43	1.96	2.45	2.94	1.48	1.92	2.37	3.43	4.28	5.14	3.92	4.89	5.87	4.28	5.57	6.85
A26	0.98	1.97	2.95	1.45	2.54	3.63	0.55	1.10	1.65	0.00	0.55	1.10	4.49	5.84	7.18	7.18	8.08	8.98	2.20	2.75	3.30	0.98	1.97	2.95	4.92	6.40	7.88
A27	0.67	1.33	2.00	3.51	4.39	5.27	0.00	0.98	1.96	3.06	3.83	4.59	1.33	2.33	3.33	1.53	2.68	3.83	1.53	2.68	3.83	0.42	0.85	1.27	1.69	2.11	2.54
A28	1.98	2.47	2.97	0.12	0.24	0.35	0.34	0.68	1.02	4.96	6.45	7.93	1.72	3.01	4.30	1.57	2.75	3.93	2.61	3.26	3.92	1.14	2.00	2.85	3.83	4.31	4.79
A29	4.58	5.96	7.33	3.67	4.58	5.50	1.97	3.46	4.94	4.58	5.96	7.33	7.33	8.25	9.17	4.21	5.48	6.74	3.67	4.58	5.50	4.94	6.42	7.90	4.21	5.48	6.74
A30	3.39	4.40	5.42	2.71	3.39	4.07	0.00	0.68	1.36	1.36	2.37	3.39	1.36	2.37	3.39	1.61	2.83	4.04	3.39	4.40	5.42	1.12	1.95	2.79	3.69	4.61	5.54

### 3.1. Z-MABAC method

In this section, we propose the Z-MABAC method to address decision-making problems in a fuzzy environment. After determining the attribute weights, the standard function value for each alternative is computed using the MABAC method, and the distance of the standard function from the boundary approximation region is defined. Once this distance is determined, the ranking of alternatives is performed, leading to the selection of the best option. We apply this method in the context of fuzzy numbers to extend its scope of application.

**Step 1: Formation of the Comprehensive Initial Decision Matrix [** **62** **]**


$$\begin{array}{cc} & {\tilde{Z}}^{\alpha }=\begin{array}{c} A\text{1}\\ A\text{2}\\ \vdots \\ Am \end{array}[\begin{array}{cccc} {\tilde{Z}}^{\alpha }\text{1}\text{1} & {\tilde{Z}}^{\alpha }\text{1}\text{2} & \ldots & {\tilde{Z}}^{\alpha }\text{1}n\\ {\tilde{Z}}^{\alpha }\text{2}\text{1} & {\tilde{Z}}^{\alpha }\text{2}\text{2} & \ldots & {\tilde{Z}}^{\alpha }\text{2}n\\ \vdots & \vdots & \ldots & \vdots \\ {\tilde{Z}}^{\alpha }m\text{1} & {\tilde{Z}}^{\alpha }m\text{2} & \ldots & {\tilde{Z}}^{\alpha }mn \end{array}]\\ & \left(i=\text{1},\text{2}\ldots m;j=\text{1},\text{2},\ldots n\right) \end{array}$$ (16)

In this Step, (Ai=1,2,…,m) denotes the set of failure modes (alternatives), and (Cj=1,2,…,n) represents the evaluation criteria corresponding to severity (S), occurrence (O), and detection (D). The term ${\tilde{Z}}_{ij}$ indicates the Z-number-based evaluation of alternative $A_{i}$ with respect to criterion $C_{j}$, incorporating both uncertainty and reliability. The decision matrix constructed in this step serves as the input for subsequent normalization and distance calculations in the Z-MABAC procedure.

Table 8 presents the comprehensive initial decision matrix used for the Z-MABAC (Multi-Attributive Border Approximation area Comparison) method. This matrix is constructed based on the ZE-number evaluations obtained from decision makers judgments. It includes risk factor assessments across multiple criteria, such as Severity (S), Occurrence (O), and Detection (D), using fuzzy values. This step serves as the foundation for ranking risk factors by normalizing and weighting the data, ultimately guiding the decision-making process in medicinal plant extraction risk analysis.

**Table 8:** Initial decision matrix for the Z-MABAC method.

	S			O			D		
A1	2.00	3.67	5.67	3.67	5.67	7.67	3.00	5.00	7.00
A2	5.67	7.67	9.33	7.00	9.00	10.00	7.67	9.00	9.67
A3	3.67	5.67	7.67	1.67	3.67	5.67	1.33	2.67	4.33
A4	1.67	3.67	5.67	3.00	5.00	7.00	3.67	5.67	7.67
A5	4.33	6.33	8.00	5.67	7.67	9.33	6.33	6.67	8.00
A6	5.00	7.00	8.67	6.33	8.00	9.33	7.00	8.33	9.00
A7	3.00	5.00	7.00	3.00	5.00	7.00	4.33	6.33	8.33
A8	4.00	5.67	7.33	4.33	6.33	8.00	2.33	4.33	6.33
A9	5.67	7.67	9.33	5.67	7.67	9.33	7.00	8.67	9.67
A10	4.33	6.33	8.33	7.67	9.00	9.67	7.67	9.00	9.67
A11	2.33	4.33	6.33	4.33	6.33	8.33	2.00	3.67	5.67
A12	0.33	1.33	3.00	2.00	3.67	5.67	1.33	2.67	4.33
A13	3.67	5.67	7.67	7.67	9.33	10.00	3.33	4.33	5.33
A14	1.67	3.67	5.67	3.00	5.00	7.00	2.67	4.33	6.00
A15	3.67	5.67	7.67	5.67	7.67	9.33	5.00	6.67	8.00
A16	1.67	3.67	5.67	1.33	3.00	5.00	3.67	5.67	7.33
A17	6.33	8.00	9.33	5.00	7.00	8.67	3.67	5.67	7.67
A18	5.33	6.67	7.67	6.33	7.67	8.33	4.67	5.67	7.33
A19	3.00	5.00	7.00	1.67	3.67	5.67	3.67	5.67	7.67
A20	0.67	2.33	4.33	2.00	3.67	5.67	1.67	3.67	5.67
A21	0.67	2.33	4.33	2.00	3.67	5.67	2.00	3.67	5.67
A22	4.33	6.33	8.33	5.67	7.33	8.67	3.67	5.67	7.67
A23	0.33	1.33	3.00	2.67	4.00	5.67	2.00	3.33	5.00
A24	1.67	3.67	5.67	2.33	4.33	6.33	2.33	4.33	6.33
A25	4.33	6.33	8.33	3.67	5.67	7.67	4.33	6.33	8.33
A26	1.00	2.00	3.67	2.00	3.67	5.67	4.67	6.00	7.33
A27	1.33	3.00	5.00	1.33	3.00	5.00	1.33	2.67	4.33
A28	3.67	5.67	7.67	0.67	2.33	4.33	3.33	4.67	6.00
A29	4.33	6.33	8.00	5.00	7.00	8.67	5.33	7.67	9.00
A30	5.00	7.00	8.67	5.00	7.00	9.00	5.67	7.67	9.33

Specifically, expert consensus is quantified through the evaluation-counting mechanism (Y,N,θ), representing agreement, disagreement, and neutrality toward the decision-makers’ initial Z-number assessments. The resulting reliability scores are reported in Table 6 and are used to adjust the reliability component during the construction of ZE-numbers.

After incorporating uncertainty, reliability, and group consensus, the ZE-number evaluations are transformed into triangular fuzzy numbers (l,m,u), forming the ZE-based fuzzy decision matrix presented in Table 7. Consequently, the MABAC procedure including normalization, weighting, border approximation area determination, and distance calculation is applied to this fuzzy decision matrix rather than directly to ZE-numbers. The normalized fuzzy decision matrix used in the ZE–MABAC analysis is shown in Table 9. Therefore, although reliability values do not appear as separate outputs within the MABAC computation steps, their effects are fully embedded in the ZE-based fuzzy inputs used by the Z-MABAC method.

**Table 9:** Normalized decision matrix obtained by scaling the initial values.

	S			O			D		
A1	0.19	0.37	0.59	0.32	0.54	0.75	0.2	0.44	0.68
A2	0.59	0.81	1.00	0.68	0.89	1.00	0.76	0.92	1
A3	0.37	0.59	0.81	0.11	0.32	0.54	0	0.16	0.36
A4	0.15	0.37	0.59	0.25	0.46	0.68	0.28	0.52	0.76
A5	0.44	0.67	0.85	0.54	0.75	0.93	0.6	0.64	0.8
A6	0.52	0.74	0.93	0.61	0.79	0.93	0.68	0.84	0.92
A7	0.30	0.52	0.74	0.25	0.46	0.68	0.36	0.6	0.84
A8	0.41	0.59	0.78	0.39	0.61	0.79	0.12	0.36	0.6
A9	0.59	0.81	1.00	0.54	0.75	0.93	0.68	0.88	1
A10	0.44	0.67	0.89	0.75	0.89	0.96	0.76	0.92	1
A11	0.22	0.44	0.67	0.39	0.61	0.82	0.08	0.28	0.52
A12	0.00	0.11	0.30	0.14	0.32	0.54	0	0.16	0.36
A13	0.37	0.59	0.81	0.75	0.93	1.00	0.24	0.36	0.48
A14	0.15	0.37	0.59	0.25	0.46	0.68	0.16	0.36	0.56
A15	0.37	0.59	0.81	0.54	0.75	0.93	0.44	0.64	0.8
A16	0.15	0.37	0.59	0.07	0.25	0.46	0.28	0.52	0.72
A17	0.67	0.85	1.00	0.46	0.68	0.86	0.28	0.52	0.76
A18	0.56	0.70	0.81	0.61	0.75	0.82	0.4	0.52	0.72
A19	0.30	0.52	0.74	0.11	0.32	0.54	0.28	0.52	0.76
A20	0.04	0.22	0.44	0.14	0.32	0.54	0.04	0.28	0.52
A21	0.04	0.22	0.44	0.14	0.32	0.54	0.08	0.28	0.52
A22	0.44	0.67	0.89	0.54	0.71	0.86	0.28	0.52	0.76
A23	0.00	0.11	0.30	0.21	0.36	0.54	0.08	0.24	0.44
A24	0.15	0.37	0.59	0.18	0.39	0.61	0.12	0.36	0.6
A25	0.44	0.67	0.89	0.32	0.54	0.75	0.36	0.6	0.84
A26	0.07	0.19	0.37	0.14	0.32	0.54	0.4	0.56	0.72
A27	0.11	0.30	0.52	0.07	0.25	0.46	0	0.16	0.36
A28	0.37	0.59	0.81	0.00	0.18	0.39	0.24	0.4	0.56
A29	0.44	0.67	0.85	0.46	0.68	0.86	0.48	0.76	0.92
A30	0.52	0.74	0.93	0.46	0.68	0.89	0.52	0.76	0.96

**Step 2: Normalization of the Comprehensive Initial Decision Matrix**

In Step 2, the initial decision matrix (Table 8) undergoes normalization to standardize the values across different criteria, ensuring a consistent scale for comparison. Normalization is essential for eliminating bias due to varying measurement units and ranges. The process transforms the values into a comparable format while preserving their relative significance. It should be noted that the normalization equation is not applied directly to ZE-numbers. In this study, ZE-number evaluations are first converted into triangular fuzzy numbers (l,m,u) after incorporating uncertainty, reliability, and group consensus. The normalization procedure is then applied separately to the lower, middle, and upper bounds of these fuzzy numbers, which makes the normalization process compatible with the fuzzy ZE-number-based framework. This step is performed using the following mathematical formula: [62].


$${\tilde{Z}}_{\text{ij}}^{\prime }=\left(\frac{Z_{\text{ij}\text{1}}}{\text{maxi}\left(Z_{\text{ij}\text{4}}\right)},\frac{Z_{\text{ij}\text{2}}}{\text{maxi}\left(Z_{\text{ij}\text{4}}\right)},\frac{Z_{\text{ij}\text{3}}}{\text{maxi}\left(Z_{\text{ij}\text{4}}\right)},\frac{Z_{\text{ij}\text{4}}}{\text{maxi}\left(Z_{\text{ij}\text{4}}\right)}\right)$$

Where the Z_ij_′ represents the normalized value of the risk factor for alternative iii and criterion j, Z_ij1_, Z_ij2_, Z_ij3_, Z_ij4_ are the original fuzzy values and maxi (Z_ij4_) is the maximum value of the fourth component across all alternatives. This normalization ensures that all values are adjusted relative to the highest observed value in the dataset, maintaining consistency and facilitating a fair comparison of risk factors in medicinal plant extraction. This formula ensures that all values fall within a defined range, allowing for an unbiased risk factor ranking in the Z-MABAC method. The normalized decision matrix obtained by scaling the initial evaluation values is presented in Table 9, which serves as the basis for subsequent weighting and risk ranking using the Z-MABAC method.

**Step 3: Calculation of the Weighted Decision Matrix**

In this step, the normalized decision matrix from Step 2 is transformed into the weighted decision matrix by incorporating the importance weights assigned to each criterion. Unlike a simple multiplication approach, this method adjusts the values by adding a constant term $\tilde{C}\;$ before applying the weights. The calculation follows this formula: [62]


$$\tilde{R}=(\begin{array}{cccc} {\tilde{r}}_{\text{1}\text{1}} & {\tilde{r}}_{\text{1}\text{2}} & \cdots & {\tilde{r}}_{\text{1}\text{n}}\\ {\tilde{r}}_{\text{2}\text{1}} & {\tilde{r}}_{\text{2}\text{2}} & \cdots & {\tilde{r}}_{\text{2}n}\\ \vdots & \vdots & \ldots & \vdots \\ {\tilde{r}}_{m\text{1}} & {\tilde{r}}_{m\text{2}} & \cdots & {\tilde{r}}_{mn} \end{array})=(\begin{array}{cccc} w_{\text{1}}\left(\tilde{C}+{\tilde{Z}}_{\text{1}\text{1}}^{\prime }\right) & w_{\text{2}}\left(\tilde{C}+{\tilde{Z}}_{\text{1}\text{2}}^{\prime }\right) & \cdots & w_{n}\left(\tilde{C}+{\tilde{Z}}_{\text{1}n}^{\prime }\right)\\ w_{\text{1}}\left(\tilde{C}+{\tilde{Z}}_{\text{2}\text{1}}^{\prime }\right) & w_{\text{2}}\left(\tilde{C}+{\tilde{Z}}_{\text{2}\text{2}}^{\prime }\right) & \cdots & w_{n}\left(\tilde{C}+{\tilde{Z}}_{\text{2}n}^{\prime }\right)\\ \vdots & \vdots & \cdots & \vdots \\ w_{\text{1}}\left(\tilde{C}+{\tilde{Z}}_{m\text{1}}^{\prime }\right) & w_{\text{2}}\left(\tilde{C}+{\tilde{Z}}_{m\text{2}}^{\prime }\right) & \cdots & w_{n}\left(\tilde{C}+{\tilde{Z}}_{mn}^{\prime }\right) \end{array})$$

Where, $\tilde{R}$ represents the weighted decision matrix, $w_{j}$ is the weight assigned to criterion j, $\tilde{Z_{IJ^{\prime }}}$′ are the normalized values from Step 2,$\tilde{C}$ is a constant value added to each normalized value before weighting. This approach ensures that weights influence the decision matrix in a structured way, maintaining the consistency of decision makers evaluations while emphasizing the importance of each criterion. Table 10 presents the weighted decision matrix obtained by adjusting the normalized values from Table 9 using assigned weights and an additional constant term. This step refines the risk prioritization by ensuring that more significant criteria have a greater impact on the final ranking.

**Table 10:** Weighted decision matrix.

	S			O			D		
A1	0.41	0.11	0.41	0.32	0.59	0.13	0.10	0.20	0.21
A2	0.55	0.14	0.52	0.40	0.72	0.15	0.15	0.27	0.25
A3	0.47	0.13	0.47	0.27	0.51	0.12	0.09	0.16	0.17
A4	0.40	0.11	0.41	0.30	0.56	0.13	0.11	0.22	0.22
A5	0.50	0.13	0.48	0.37	0.67	0.15	0.14	0.23	0.23
A6	0.52	0.14	0.50	0.39	0.68	0.15	0.14	0.26	0.24
A7	0.45	0.12	0.45	0.30	0.56	0.13	0.12	0.23	0.23
A8	0.49	0.13	0.46	0.33	0.61	0.13	0.10	0.19	0.20
A9	0.55	0.14	0.52	0.37	0.67	0.15	0.14	0.27	0.25
A10	0.50	0.13	0.49	0.42	0.72	0.15	0.15	0.27	0.25
A11	0.42	0.11	0.43	0.33	0.61	0.14	0.09	0.18	0.19
A12	0.35	0.09	0.34	0.27	0.51	0.12	0.09	0.16	0.17
A13	0.47	0.13	0.47	0.42	0.74	0.15	0.11	0.19	0.19
A14	0.40	0.11	0.41	0.30	0.56	0.13	0.10	0.19	0.20
A15	0.47	0.13	0.47	0.37	0.67	0.15	0.12	0.23	0.23
A16	0.40	0.11	0.41	0.26	0.48	0.11	0.11	0.22	0.22
A17	0.58	0.15	0.52	0.35	0.64	0.14	0.11	0.22	0.22
A18	0.54	0.14	0.47	0.39	0.67	0.14	0.12	0.22	0.22
A19	0.45	0.12	0.45	0.27	0.51	0.12	0.11	0.22	0.22
A20	0.36	0.10	0.37	0.27	0.51	0.12	0.09	0.18	0.19
A21	0.36	0.10	0.37	0.27	0.51	0.12	0.09	0.18	0.19
A22	0.50	0.13	0.49	0.37	0.66	0.14	0.11	0.22	0.22
A23	0.35	0.09	0.34	0.29	0.52	0.12	0.09	0.18	0.18
A24	0.40	0.11	0.41	0.28	0.53	0.12	0.10	0.19	0.20
A25	0.50	0.13	0.49	0.32	0.59	0.13	0.12	0.23	0.23
A26	0.37	0.09	0.35	0.27	0.51	0.12	0.12	0.22	0.22
A27	0.38	0.10	0.39	0.26	0.48	0.11	0.09	0.16	0.17
A28	0.47	0.13	0.47	0.24	0.45	0.10	0.11	0.20	0.20
A29	0.50	0.13	0.48	0.35	0.64	0.14	0.13	0.25	0.24
A30	0.52	0.14	0.50	0.35	0.64	0.14	0.13	0.25	0.25

**Step 4: Calculation of the Border Approximation Area (BAA) Values**

In Step 4, the Border Approximation Area (BAA) values are calculated as part of the Z-MABAC method. This step determines the ideal decision region by computing the border approximation area for each criterion. The BAA values serve as reference points, allowing the differentiation between favorable and unfavorable alternatives. The BAA values are computed using the following formula: [62]


$${\tilde{g}}_{j}={\left(\prod_{i=\text{1}}^{m}\;{\tilde{r}}_{ij}\right)}^{\frac{\text{1}}{m}}\;\tilde{G}=\left({\tilde{g}}_{\text{1}},{\tilde{g}}_{\text{2}},\cdots ,{\tilde{g}}_{n}\right)$$

Where Q ~ i represents the utility function value for alternative I, $\tilde{R_{ij}}$ is the weighted decision matrix value for alternative iii and criterion j, is the Border Approximation Area value for criterion j, and n is the total number of criteria. A higher $\tilde{G_{j}}$ Value indicates a more favorable alternative, meaning the risk factor is more significant. The alternatives are ranked based on these values, allowing for informed decision-making in risk assessment.

**Step 5: Calculation of the Alternative Utility Function Values**

Calculate the Distance Between Each Alternative and the Border Approximation Area for the Matrix Elements. In this step, the final ranking of alternatives is determined by computing the Alternative Utility Function values ($\tilde{Q}$). This calculation evaluates how each alternative deviates from the BAA values ($\tilde{G}$), allowing for an accurate ranking of risk factors in medicinal plant extraction. The Alternative Utility Function matrix $\tilde{Q}$ is computed using the following formula: [62]


$$\begin{array}{c} \tilde{Q}=\tilde{R}-\tilde{G} \end{array}$$ (17)

$$\tilde{Q}=(\begin{array}{cccc} {\tilde{q}}_{\text{1}\text{1}} & {\tilde{q}}_{\text{1}\text{2}} & \cdots & {\tilde{q}}_{\text{1}n}\\ {\tilde{q}}_{\text{2}\text{1}} & {\tilde{q}}_{\text{2}\text{2}} & \cdots & {\tilde{q}}_{\text{2}n}\\ \vdots & \vdots & \ldots & \vdots \\ {\tilde{q}}_{m\text{1}} & {\tilde{q}}_{m\text{2}} & \cdots & {\tilde{q}}_{mn} \end{array})$$ (18)

Where $\tilde{Q}$ represents the matrix of utility function values, $\tilde{R}$ is the weighted decision matrix obtained in Step 3 and $\tilde{G}$ is the Border Approximation Area matrix from Step 4. The expanded form of this equation is: [62]


$$\begin{array}{c} \tilde{Q}=\tilde{R}-\tilde{G\;}=(\begin{array}{cccc} {\tilde{r}}_{\text{1}\text{1}} & {\tilde{r}}_{\text{1}\text{2}} & \ldots & {\tilde{r}}_{\text{1}n}\\ {\tilde{r}}_{\text{2}\text{1}} & {\tilde{r}}_{\text{2}\text{2}} & \ldots & {\tilde{r}}_{\text{2}n}\\ \vdots & \vdots & \ldots & \vdots \\ {\tilde{r}}_{m\text{1}} & {\tilde{r}}_{m\text{2}} & \ldots & {\tilde{r}}_{mn} \end{array})-(\begin{array}{cccc} {\tilde{g}}_{\text{1}} & {\tilde{g}}_{\text{2}} & \ldots & {\tilde{g}}_{n}\\ {\tilde{g}}_{\text{1}} & {\tilde{g}}_{\text{2}} & \ldots & {\tilde{g}}_{n}\\ \vdots & \vdots & \ldots & \vdots \\ {\tilde{g}}_{\text{1}} & {\tilde{g}}_{\text{2}} & \ldots & {\tilde{g}}_{n} \end{array}) \end{array}$$ (19)

**Interpretation of Utility Function Values:**

- If $\tilde{Q_{ij}}>\text{0}$, the alternative iii performs better than the ideal decision boundary, meaning it has a higher significance in risk prioritization.
- If $\tilde{Q_{ij}}<\text{0}$, the alternative iii is below the decision boundary, indicating a lower significance in the decision-making process.

The alternatives are ranked based on these utility function values, with higher values signifying greater importance in risk assessment. This step provides the final ranking of risk factors, helping decision-makers in prioritizing key risks in medicinal plant extraction. Table 11 presents the final utility function values ($\tilde{Q}$) calculated using the Z-MABAC method. These values represent the deviation of each alternative from the BAA, allowing for the prioritization of risk factors in medicinal plant extraction. Higher utility values indicate greater significance, while lower or negative values suggest less critical risks. The ranking derived from this table provides a structured approach to decision-making, ensuring that the most influential risks are addressed first.

**Table 11:** Distance Matrix for the Border Approximation Area.

	S			O			D		
A1	−0.03	−0.01	−0.04	0.19	0.00	−0.19	−0.11	−0.01	0.10
A2	0.11	0.03	0.07	0.27	0.14	−0.17	−0.06	0.06	0.14
A3	0.03	0.01	0.02	0.14	−0.08	−0.20	−0.13	−0.05	0.06
A4	−0.04	−0.01	−0.04	0.17	−0.02	−0.19	−0.10	0.00	0.11
A5	0.06	0.01	0.03	0.24	0.09	−0.17	−0.08	0.02	0.12
A6	0.08	0.02	0.05	0.26	0.10	−0.17	−0.07	0.05	0.13
A7	0.01	0.00	0.00	0.17	−0.02	−0.19	−0.10	0.02	0.12
A8	0.04	0.01	0.01	0.21	0.03	−0.19	−0.12	−0.02	0.09
A9	0.11	0.03	0.07	0.24	0.09	−0.17	−0.07	0.06	0.14
A10	0.06	0.01	0.04	0.29	0.14	−0.17	−0.06	0.06	0.14
A11	−0.02	0.00	−0.02	0.21	0.03	−0.18	−0.12	−0.03	0.08
A12	−0.10	−0.03	−0.11	0.15	−0.08	−0.20	−0.13	−0.05	0.06
A13	0.03	0.01	0.02	0.29	0.15	−0.17	−0.11	−0.02	0.08
A14	−0.04	−0.01	−0.04	0.17	−0.02	−0.19	−0.11	−0.02	0.09
A15	0.03	0.01	0.02	0.24	0.09	−0.17	−0.09	0.02	0.12
A16	−0.04	−0.01	−0.04	0.13	−0.11	−0.21	−0.10	0.00	0.11
A17	0.13	0.03	0.07	0.22	0.06	−0.18	−0.10	0.00	0.11
A18	0.10	0.02	0.02	0.26	0.09	−0.18	−0.09	0.00	0.11
A19	0.01	0.00	0.00	0.14	−0.08	−0.20	−0.10	0.00	0.11
A20	−0.08	−0.02	−0.08	0.15	−0.08	−0.20	−0.12	−0.03	0.08
A21	−0.08	−0.02	−0.08	0.15	−0.08	−0.20	−0.12	−0.03	0.08
A22	0.06	0.01	0.04	0.24	0.07	−0.18	−0.10	0.00	0.11
A23	−0.10	−0.03	−0.11	0.16	−0.07	−0.20	−0.12	−0.04	0.07
A24	−0.04	−0.01	−0.04	0.15	−0.05	−0.20	−0.12	−0.02	0.09
A25	0.06	0.01	0.04	0.19	0.00	−0.19	−0.10	0.02	0.12
A26	−0.07	−0.03	−0.09	0.15	−0.08	−0.20	−0.09	0.01	0.11
A27	−0.06	−0.02	−0.06	0.13	−0.11	−0.21	−0.13	−0.05	0.06
A28	0.03	0.01	0.02	0.11	−0.13	−0.21	−0.11	−0.01	0.09
A29	0.06	0.01	0.03	0.22	0.06	−0.18	−0.09	0.04	0.13
A30	0.08	0.02	0.05	0.22	0.06	−0.18	−0.08	0.04	0.14

**Step 6: Final Score Calculation and Ranking of Alternatives**

In this step, the final score for each alternative is determined by summing the utility function values ($\tilde{q_{ij}})$ Across all criteria. This provides a total score that reflects the overall significance of each alternative in the decision-making process. The total score for each alternative is calculated using the following formula. [62]


$${\tilde{S}}_{i}=\sum {\mathrm{\backslash nolimits}}_{j=\text{1}}^{n}\;{\tilde{q}}_{ij},\text{ i}=\text{1},\text{2},\ldots \text{m }$$ (20)

Where $\;{\tilde{S}}_{i}$ represents the total utility score for alternative i, $\tilde{q_{ij}}$ is the utility function value for alternative iii under criterion j, n is the total number of criteria, and m is the total number of alternatives.

### 3.2. Defuzzification Process and Ranking the Alternatives.

Since the calculations involve fuzzy numbers, the total score $S_{\left(\text{1})\right)}$ must be defuzzified to obtain a precise numerical value. This process converts the fuzzy values into a crisp number, making it possible to rank the alternatives objectively. Defuzzification is performed using an appropriate method (e.g., the centroid method, mean of maxima, or another defuzzification technique). The resulting numerical values allow direct comparison of alternatives. Once the defuzzified scores are obtained, the alternatives are ranked in descending order:


$$S_{\left(\text{1}\right)}\geq S_{\left(\text{2}\right)}\geq \cdots \geq S_{\left(m\right)}$$ (21)

where higher scores indicate alternatives with greater significance in risk evaluation. This step provides the final ranking of risk factors, enabling informed decision-making in medicinal plant extraction. Table 12 presents the final ranking of alternatives based on the total utility scores obtained in Step 6. The table includes the aggregated and defuzzifiedscores for each alternative, allowing for an objective comparison of risk factors in medicinal plant extraction. Higher scores indicate more critical risks, while lower scores correspond to less significant ones. This ranking provides decision-makers with a structured approach to prioritizing key risk factors for mitigation.

**Table 12:** Final ranking of risk factors in medicinal plant extraction based on defuzzified utility scores.

	Si			Defuzzification of Si	Ranking
A1	−0.18	−0.01	0.11	−0.03	17
A2	0.09	0.23	0.27	0.20	1
A3	−0.19	−0.12	0.11	−0.07	21
A4	−0.21	−0.03	0.11	−0.04	19
A5	−0.01	0.12	0.20	0.10	7
A6	0.04	0.17	0.24	0.15	4
A7	−0.15	−0.01	0.16	0.00	15
A8	−0.10	0.02	0.15	0.02	14
A9	0.05	0.17	0.27	0.16	3
A10	0.06	0.21	0.24	0.17	2
A11	−0.17	0.00	0.11	−0.02	16
A12	−0.31	−0.16	−0.03	−0.16	30
A13	−0.01	0.14	0.16	0.10	9
A14	−0.22	−0.05	0.09	−0.06	20
A15	−0.05	0.11	0.19	0.09	11
A16	−0.25	−0.11	0.09	−0.09	24
A17	0.02	0.09	0.23	0.12	6
A18	0.03	0.11	0.18	0.10	8
A19	−0.19	−0.07	0.14	−0.04	18
A20	−0.29	−0.13	0.03	−0.13	27
A21	−0.29	−0.13	0.03	−0.13	26
A22	−0.04	0.09	0.20	0.09	12
A23	−0.28	−0.13	−0.01	−0.14	29
A24	−0.24	−0.08	0.09	−0.08	23
A25	−0.08	0.03	0.21	0.05	13
A26	−0.25	−0.09	0.04	−0.10	25
A27	−0.29	−0.17	0.03	−0.14	28
A28	−0.19	−0.14	0.12	−0.07	22
A29	−0.04	0.11	0.21	0.10	10
A30	−0.01	0.12	0.24	0.12	5

### 3.3. Development of the MABAC Method in the ZE-Numbers Environment

The MABAC method is a well-established multi-criteria decision-making approach that enables the ranking of alternatives based on their distance from a border approximation area. Recent studies have extended the MABAC method under the framework of ZE-numbers to explicitly account for uncertainty, expert reliability, and group consensus in decision-making processes [63]. In these extensions, ZE-numbers enhance conventional fuzzy logic by incorporating a reliability component, thereby providing a more structured and realistic representation of expert judgments.

In line with these developments, the present study adopts the ZE-MABAC formulation proposed in the literature and applies it within a unified risk assessment framework for medicinal plant extraction. The methodological novelty of this research does not lie in proposing a new ZE-MABAC model, but rather in its systematic integration with FMEA and ZE-based criteria weighting to address a real-world extraction process characterized by uncertainty and heterogeneous expert opinions.

Moreover, the weighting of risk criteria is conducted using the ZE-BWM approach, following the extended ZE-number framework introduced by Haseli et al. [64]. By employing ZE-numbers at the data elicitation stage and subsequently transforming them into triangular fuzzy numbers, the proposed framework ensures compatibility with ZE-BWM and ZE-MABAC while preserving information related to expert reliability and group consensus. As a result, the prioritization of risk factors becomes more robust and transparent, reflecting both the severity of failures and the credibility of expert assessments.

**Step 1: Constructing the Comprehensive Decision Matrix.**

The first step in applying the MABAC method within the ZE-numbers environment is constructing the comprehensive decision matrix. This matrix is developed based on expert evaluations, where risk factors are assessed using linguistic terms that are converted into ZE-numbers. Each row in the matrix represents an alternative (risk factor), while each column corresponds to a decision criterion. The inclusion of ZE-numbers ensures that expert opinions are quantified while maintaining a degree of uncertainty and reliability in decision-making. This matrix serves as the foundation for further computations in the MABAC method, facilitating a structured and accurate risk prioritization process. Table 13 presents the initial decision matrix constructed using expert evaluations in the ZE-numbers environment. Each entry in the table consists of a ZE-number, which includes both a fuzzy value representing the expert’s assessment and a reliability parameter indicating confidence in the evaluation. This matrix provides a structured representation of the decision-making problem and serves as the basis for the subsequent normalization and weighting steps in the MABAC method.

**Table 13:** The initial decision matrix is formed based on experts’ opinions.

	S			O			D		
A1	2.00	3.67	5.67	3.67	5.67	7.67	3.00	5.00	7.00
A2	5.67	7.67	9.33	7.00	9.00	10.00	7.67	9.00	9.67
A3	3.67	5.67	7.67	1.67	3.67	5.67	1.33	2.67	4.33
A4	1.67	3.67	5.67	3.00	5.00	7.00	3.67	5.67	7.67
A5	4.33	6.33	8.00	5.67	7.67	9.33	6.33	6.67	8.00
A6	5.00	7.00	8.67	6.33	8.00	9.33	7.00	8.33	9.00
A7	3.00	5.00	7.00	3.00	5.00	7.00	4.33	6.33	8.33
A8	4.00	5.67	7.33	4.33	6.33	8.00	2.33	4.33	6.33
A9	5.67	7.67	9.33	5.67	7.67	9.33	7.00	8.67	9.67
A10	4.33	6.33	8.33	7.67	9.00	9.67	7.67	9.00	9.67
A11	2.33	4.33	6.33	4.33	6.33	8.33	2.00	3.67	5.67
A12	0.33	1.33	3.00	2.00	3.67	5.67	1.33	2.67	4.33
A13	3.67	5.67	7.67	7.67	9.33	10.00	3.33	4.33	5.33
A14	1.67	3.67	5.67	3.00	5.00	7.00	2.67	4.33	6.00
A15	3.67	5.67	7.67	5.67	7.67	9.33	5.00	6.67	8.00
A16	1.67	3.67	5.67	1.33	3.00	5.00	3.67	5.67	7.33
A17	6.33	8.00	9.33	5.00	7.00	8.67	3.67	5.67	7.67
A18	5.33	6.67	7.67	6.33	7.67	8.33	4.67	5.67	7.33
A19	3.00	5.00	7.00	1.67	3.67	5.67	3.67	5.67	7.67
A20	0.67	2.33	4.33	2.00	3.67	5.67	1.67	3.67	5.67
A21	0.67	2.33	4.33	2.00	3.67	5.67	2.00	3.67	5.67
A22	4.33	6.33	8.33	5.67	7.33	8.67	3.67	5.67	7.67
A23	0.33	1.33	3.00	2.67	4.00	5.67	2.00	3.33	5.00
A24	1.67	3.67	5.67	2.33	4.33	6.33	2.33	4.33	6.33
A25	4.33	6.33	8.33	3.67	5.67	7.67	4.33	6.33	8.33
A26	1.00	2.00	3.67	2.00	3.67	5.67	4.67	6.00	7.33
A27	1.33	3.00	5.00	1.33	3.00	5.00	1.33	2.67	4.33
A28	3.67	5.67	7.67	0.67	2.33	4.33	3.33	4.67	6.00
A29	4.33	6.33	8.00	5.00	7.00	8.67	5.33	7.67	9.00
A30	5.00	7.00	8.67	5.00	7.00	9.00	5.67	7.67	9.33

**Step 2: Normalizing the Comprehensive Decision Matrix.**

After constructing the initial decision matrix, the next step in the MABAC method within the ZE-numbers environment is normalization. The normalization process ensures that all values across different criteria are transformed into a comparable scale, eliminating inconsistencies caused by varying measurement units. Since the decision matrix contains ZE-numbers, the normalization process must preserve both the fuzzy and reliability components of the data. By normalizing the decision matrix, this step allows for a structured comparison of alternatives, preparing the data for the weighting and ranking processes in the MABAC method. Table 14 presents the normalized decision matrix, where the initial ZE-number evaluations from Table 13 have been transformed to a standardized scale. The normalization ensures that all risk factor assessments are comparable across different criteria. Each entry in Table 14 represents a normalized ZE-number, maintaining the balance between fuzzy assessments and expert reliability while standardizing the values for the next steps in the MABAC method. This transformation ensures that the decision-making process remains objective and accurate.

**Table 14:** Normalized decision matrix in the ZE-numbers.

	S			O			D		
A1	0.19	0.37	0.59	0.32	0.54	0.75	0.2	0.44	0.68
A2	0.59	0.81	1.00	0.68	0.89	1.00	0.76	0.92	1
A3	0.37	0.59	0.81	0.11	0.32	0.54	0	0.16	0.36
A4	0.15	0.37	0.59	0.25	0.46	0.68	0.28	0.52	0.76
A5	0.44	0.67	0.85	0.54	0.75	0.93	0.6	0.64	0.8
A6	0.52	0.74	0.93	0.61	0.79	0.93	0.68	0.84	0.92
A7	0.30	0.52	0.74	0.25	0.46	0.68	0.36	0.6	0.84
A8	0.41	0.59	0.78	0.39	0.61	0.79	0.12	0.36	0.6
A9	0.59	0.81	1.00	0.54	0.75	0.93	0.68	0.88	1
A10	0.44	0.67	0.89	0.75	0.89	0.96	0.76	0.92	1
A11	0.22	0.44	0.67	0.39	0.61	0.82	0.08	0.28	0.52
A12	0.00	0.11	0.30	0.14	0.32	0.54	0	0.16	0.36
A13	0.37	0.59	0.81	0.75	0.93	1.00	0.24	0.36	0.48
A14	0.15	0.37	0.59	0.25	0.46	0.68	0.16	0.36	0.56
A15	0.37	0.59	0.81	0.54	0.75	0.93	0.44	0.64	0.8
A16	0.15	0.37	0.59	0.07	0.25	0.46	0.28	0.52	0.72
A17	0.67	0.85	1.00	0.46	0.68	0.86	0.28	0.52	0.76
A18	0.56	0.70	0.81	0.61	0.75	0.82	0.4	0.52	0.72
A19	0.30	0.52	0.74	0.11	0.32	0.54	0.28	0.52	0.76
A20	0.04	0.22	0.44	0.14	0.32	0.54	0.04	0.28	0.52
A21	0.04	0.22	0.44	0.14	0.32	0.54	0.08	0.28	0.52
A22	0.44	0.67	0.89	0.54	0.71	0.86	0.28	0.52	0.76
A23	0.00	0.11	0.30	0.21	0.36	0.54	0.08	0.24	0.44
A24	0.15	0.37	0.59	0.18	0.39	0.61	0.12	0.36	0.6
A25	0.44	0.67	0.89	0.32	0.54	0.75	0.36	0.6	0.84
A26	0.07	0.19	0.37	0.14	0.32	0.54	0.4	0.56	0.72
A27	0.11	0.30	0.52	0.07	0.25	0.46	0	0.16	0.36
A28	0.37	0.59	0.81	0.00	0.18	0.39	0.24	0.4	0.56
A29	0.44	0.67	0.85	0.46	0.68	0.86	0.48	0.76	0.92
A30	0.52	0.74	0.93	0.46	0.68	0.89	0.52	0.76	0.96

**Step 3: Calculating the Elements of the Weighted Matrix.**

Step three in the MABAC method within the ZE-numbers environment involves computing the weighted decision matrix, where the normalized values from Table 14 are adjusted based on their respective criterion weights. This ensures that more critical factors exert a stronger influence on the decision-making process. Each criterion is assigned a weight (wj), reflecting its significance in evaluating risk factors, and the weighted values are calculated using the formula:


$${\tilde{R}}_{ij}=w_{j}\times {\tilde{Z}}_{ij}^{\prime }$$

where $\tilde{R_{ij}}$ represents the weighted decision matrix value, wj is the assigned weight, and Z ~ ij′ is the normalized value. By integrating expert reliability and weighting the criteria appropriately, this step refines the decision-making process, ensuring that key risk factors are accurately prioritized. Table 15 presents the weighted decision matrix, where normalized values have been transformed based on their respective importance weights. Each entry represents a weighted ZE-number, preserving both expert reliability and the structured evaluation of risk factors. This table plays a crucial role in further computations within the MABAC method, forming the foundation for ranking risk factors in medicinal plant extraction.

**Table 15:** Weighted decision matrix.

	S			O			D		
A1	0.33	0.81	0.26	0.17	0.37	0.93	0.72	0.24	0.51
A2	0.44	1.07	0.33	0.21	0.46	1.06	1.05	0.32	0.60
A3	0.38	0.94	0.30	0.14	0.32	0.81	0.60	0.19	0.41
A4	0.32	0.81	0.26	0.16	0.36	0.89	0.76	0.25	0.53
A5	0.40	0.98	0.31	0.19	0.43	1.02	0.96	0.27	0.54
A6	0.42	1.02	0.32	0.20	0.43	1.02	1.00	0.31	0.58
A7	0.36	0.89	0.29	0.16	0.36	0.89	0.81	0.27	0.56
A8	0.39	0.94	0.30	0.17	0.39	0.95	0.67	0.23	0.48
A9	0.44	1.07	0.33	0.19	0.43	1.02	1.00	0.31	0.60
A10	0.40	0.98	0.31	0.22	0.46	1.04	1.05	0.32	0.60
A11	0.34	0.85	0.28	0.17	0.39	0.96	0.64	0.21	0.46
A12	0.28	0.65	0.22	0.14	0.32	0.81	0.60	0.19	0.41
A13	0.38	0.94	0.30	0.22	0.47	1.06	0.74	0.23	0.45
A14	0.32	0.81	0.26	0.16	0.36	0.89	0.69	0.23	0.47
A15	0.38	0.94	0.30	0.19	0.43	1.02	0.86	0.27	0.54
A16	0.32	0.81	0.26	0.13	0.30	0.78	0.76	0.25	0.52
A17	0.46	1.09	0.33	0.18	0.41	0.98	0.76	0.25	0.53
A18	0.43	1.00	0.30	0.20	0.43	0.96	0.84	0.25	0.52
A19	0.36	0.89	0.29	0.14	0.32	0.81	0.76	0.25	0.53
A20	0.29	0.72	0.24	0.14	0.32	0.81	0.62	0.21	0.46
A21	0.29	0.72	0.24	0.14	0.32	0.81	0.64	0.21	0.46
A22	0.40	0.98	0.31	0.19	0.42	0.98	0.76	0.25	0.53
A23	0.28	0.65	0.22	0.15	0.33	0.81	0.64	0.21	0.43
A24	0.32	0.81	0.26	0.15	0.34	0.85	0.67	0.23	0.48
A25	0.40	0.98	0.31	0.17	0.37	0.93	0.81	0.27	0.56
A26	0.30	0.70	0.23	0.14	0.32	0.81	0.84	0.26	0.52
A27	0.31	0.76	0.25	0.13	0.30	0.78	0.60	0.19	0.41
A28	0.38	0.94	0.30	0.13	0.29	0.74	0.74	0.23	0.47
A29	0.40	0.98	0.31	0.18	0.41	0.98	0.88	0.29	0.58
A30	0.42	1.02	0.32	0.18	0.41	1.00	0.91	0.29	0.59

**Step 4: Calculating the Elements of the Substitution Distance Matrix for the Approximate Boundary Range (Q).**

In Step 4 of the MABAC method within the ZE-numbers environment, the Substitution Distance Matrix (Q) is calculated to measure the deviation of each alternative from the Border Approximation Area (BAA) values. This step determines the relative positioning of each alternative concerning the ideal decision boundary, allowing for the differentiation between favorable and unfavorable alternatives. The substitution distances are derived by computing the difference between the weighted decision matrix values and the BAA values for each criterion. These values help to assess whether a given alternative falls above, below, or aligns with the boundary approximation, influencing its ranking in the decision-making process. Table 16 presents the Substitution Distance Matrix (Q), showing the calculated distances between each alternative and the Border Approximation Area (BAA) across multiple criteria. Positive values indicate that an alternative performs better than the decision boundary, while negative values suggest it falls below the threshold, indicating a weaker performance. This matrix is essential for ranking alternatives effectively in the medicinal plant extraction risk assessment process.

**Table 16:** Substitution Distance Matrix (Q) measuring deviations from the Border Approximation Area (BAA) for each risk factor in medicinal plant extraction.

	S			O			D		
A1	0.04	−0.08	−0.09	−0.74	0.00	0.76	0.21	−0.01	−0.26
A2	0.16	0.19	−0.03	−0.70	0.09	0.89	0.54	0.07	−0.17
A3	0.10	0.05	−0.06	−0.77	−0.05	0.65	0.09	−0.05	−0.36
A4	0.03	−0.08	−0.09	−0.75	−0.02	0.72	0.26	0.01	−0.24
A5	0.12	0.10	−0.05	−0.72	0.05	0.85	0.45	0.03	−0.23
A6	0.14	0.14	−0.04	−0.71	0.06	0.85	0.49	0.06	−0.19
A7	0.07	0.01	−0.07	−0.75	−0.02	0.72	0.30	0.02	−0.21
A8	0.11	0.05	−0.06	−0.73	0.02	0.78	0.16	−0.02	−0.29
A9	0.16	0.19	−0.03	−0.72	0.05	0.85	0.49	0.07	−0.17
A10	0.12	0.10	−0.05	−0.69	0.09	0.87	0.54	0.07	−0.17
A11	0.05	−0.03	−0.08	−0.73	0.02	0.80	0.14	−0.03	−0.31
A12	−0.01	−0.23	−0.14	−0.77	−0.05	0.65	0.09	−0.05	−0.36
A13	0.10	0.05	−0.06	−0.69	0.10	0.89	0.23	−0.02	−0.32
A14	0.03	−0.08	−0.09	−0.75	−0.02	0.72	0.18	−0.02	−0.30
A15	0.10	0.05	−0.06	−0.72	0.05	0.85	0.35	0.03	−0.23
A16	0.03	−0.08	−0.09	−0.77	−0.07	0.61	0.26	0.01	−0.25
A17	0.18	0.21	−0.03	−0.73	0.04	0.82	0.26	0.01	−0.24
A18	0.15	0.12	−0.06	−0.71	0.05	0.80	0.33	0.01	−0.25
A19	0.07	0.01	−0.07	−0.77	−0.05	0.65	0.26	0.01	−0.24
A20	0.00	−0.16	−0.12	−0.77	−0.05	0.65	0.11	−0.03	−0.31
A21	0.00	−0.16	−0.12	−0.77	−0.05	0.65	0.14	−0.03	−0.31
A22	0.12	0.10	−0.05	−0.72	0.05	0.82	0.26	0.01	−0.24
A23	−0.01	−0.23	−0.14	−0.76	−0.04	0.65	0.14	−0.04	−0.33
A24	0.03	−0.08	−0.09	−0.76	−0.03	0.68	0.16	−0.02	−0.29
A25	0.12	0.10	−0.05	−0.74	0.00	0.76	0.30	0.02	−0.21
A26	0.01	−0.18	−0.13	−0.77	−0.05	0.65	0.33	0.01	−0.25
A27	0.02	−0.12	−0.11	−0.77	−0.07	0.61	0.09	−0.05	−0.36
A28	0.10	0.05	−0.06	−0.78	−0.08	0.57	0.23	−0.01	−0.30
A29	0.12	0.10	−0.05	−0.73	0.04	0.82	0.38	0.05	−0.19
A30	0.14	0.14	−0.04	−0.73	0.04	0.84	0.40	0.05	−0.18

**Step 5: Ranking the Options.**

In Step 5 of the MABAC method within the ZE-numbers environment, the final ranking of alternatives is determined by aggregating the elements of the Substitution Distance Matrix (Q). This step involves summing the distance values across all criteria for each alternative to compute a total performance score. The resulting scores indicate the overall significance of each alternative, with higher scores signifying greater importance in the risk assessment framework. To ensure accurate decision-making, the aggregated values are then defuzzified, converting fuzzy data into crisp numerical values that facilitate precise ranking. This ranking allows for an objective comparison of risk factors in the medicinal plant extraction process, helping prioritize key risks for mitigation. Table 17 presents the final ranking of alternatives, showing the aggregated substitution distances, their defuzzified values, and the corresponding ranking positions. Alternatives with higher scores are ranked as more critical risk factors, while lower scores indicate less significant risks. This structured ranking provides a data-driven approach to prioritizing challenges in medicinal plant extraction, ensuring that the most influential risks are addressed first. Among the identified risks, sample-related issues, extraction method inefficiencies, and instrumental errors stand out as the most influential. Sample variability and contamination (A2, A9, A10) ranked among the highest risks, indicating that inconsistencies in plant material quality, improper handling, and environmental contamination significantly impact extraction outcomes. These factors lead to variations in bioactive compound concentrations, affecting the reproducibility and potency of medicinal extracts. Table 17 extends the results reported in Table 12 by incorporating group consensus through ZE-numbers, allowing a comparison between Z-MABAC and ZE-MABAC outcomes.

**Table 17:** Final ranking of risk factors in medicinal plant extraction based on aggregated and defuzzified substitution distance values.

	Si			Defuzzification of Si	Ranking
A1	−0.72	−0.08	0.63	−0.06	18
A2	−0.23	0.35	0.93	0.35	1
A3	−0.82	−0.05	0.46	−0.14	25
A4	−0.69	−0.09	0.62	−0.05	17
A5	−0.38	0.18	0.81	0.20	6
A6	−0.31	0.26	0.86	0.27	4
A7	−0.60	0.01	0.67	0.03	14
A8	−0.70	0.05	0.66	0.00	15
A9	−0.30	0.31	0.89	0.30	2
A10	−0.26	0.26	0.89	0.30	3
A11	−0.77	−0.05	0.64	−0.06	19
A12	−0.91	−0.33	0.37	−0.29	30
A13	−0.59	0.13	0.74	0.09	13
A14	−0.76	−0.11	0.56	−0.11	21
A15	−0.50	0.13	0.80	0.15	10
A16	−0.71	−0.14	0.49	−0.12	22
A17	−0.52	0.25	0.78	0.17	8
A18	−0.46	0.18	0.72	0.15	9
A19	−0.67	−0.03	0.57	−0.04	16
A20	−0.88	−0.25	0.45	−0.23	27
A21	−0.86	−0.25	0.45	−0.22	26
A22	−0.57	0.15	0.76	0.11	11
A23	−0.86	−0.31	0.40	−0.26	29
A24	−0.80	−0.13	0.53	−0.13	24
A25	−0.55	0.12	0.73	0.10	12
A26	−0.65	−0.22	0.50	−0.13	23
A27	−0.89	−0.24	0.37	−0.25	28
A28	−0.69	−0.04	0.45	−0.10	20
A29	−0.46	0.18	0.81	0.17	7
A30	−0.42	0.22	0.85	0.22	5

## 4. Discussion

The findings from the Z-MABAC method within the ZE-numbers environment highlight the most critical failure modes in the medicinal plant extraction process. Among the identified risks, sample-related issues, extraction method inefficiencies, and instrumental errors stand out as the most influential. Sample variability and contamination (A2, A9, A10) ranked among the highest risks, indicating that inconsistencies in plant material quality, improper handling, and environmental contamination significantly impact extraction outcomes. These factors lead to variations in bioactive compound concentrations, affecting the reproducibility and potency of medicinal extracts. Addressing these failures requires strict raw material selection protocols, standardized sample preparation techniques, and improved handling practices to reduce contamination and variability.

Additionally, suboptimal extraction conditions (A6, A30) and instrumental errors (A5, A29) were identified as major risks affecting extraction efficiency and product quality. Inappropriate solvent selection, inadequate temperature and pH control, and instrumental calibration issues can result in low extraction yields, degradation of bioactive compounds, and unreliable analytical results. Ensuring regular instrument calibration, method validation, and process optimization is crucial for maintaining extraction efficiency. These failures emphasize the importance of process standardization, continuous monitoring, and personnel training to enhance extraction reliability, reduce variability, and improve overall product quality in medicinal plant extraction.

Compared with the conventional FMEA ranking based on the Risk Priority Number (RPN), the proposed Z-MABAC and ZE-MABAC approaches provide a more structured and reliable prioritization of failure modes by incorporating criteria weighting and uncertainty modeling. While classical FMEA treats severity, occurrence, and detection as independent and equally weighted factors, the proposed framework allows their relative importance to be determined through ZE-BWM, resulting in a more flexible and realistic evaluation. Furthermore, the comparison between Z-MABAC and ZE-MABAC results highlights the effect of incorporating expert reliability and group consensus into the ranking process. Although the overall pattern of critical risks remains consistent across both approaches, the ZE-MABAC method produces more robust rankings by adjusting the outcomes according to consensus levels derived from expert voting. This comparison can be regarded as a qualitative robustness check, demonstrating that the proposed framework yields stable yet more reliable results when expert reliability is explicitly considered.

### 4.1. Proposed Solutions for Risk Mitigation.

To enhance the reliability and efficiency of medicinal plant extraction, a multi-faceted risk mitigation approach is essential. First, standardizing raw material selection and sample handling through strict quality control protocols can minimize contamination and inconsistencies in bioactive compound concentrations. Second, optimizing extraction conditions by selecting appropriate solvents, temperatures, and extraction techniques tailored to specific plant metabolites can improve yield and purity. Third, ensuring instrumental accuracy through regular calibration, maintenance, and real-time monitoring systems can reduce measurement errors and enhance data reliability. Additionally, integrating AI-driven predictive models can help identify potential failures in real-time, enabling proactive risk management. Lastly, comprehensive personnel training and process automation can minimize human errors, ensuring consistency and repeatability in extraction procedures. By implementing these solutions, the medicinal plant extraction process can achieve greater standardization, higher efficiency, and improved product quality, ultimately enhancing the safety and efficacy of bioactive compounds used in pharmaceuticals and herbal medicines.

## 5. Conclusion and Further Study

The extraction of bioactive compounds from medicinal plants is a crucial process in pharmaceutical and herbal medicine industries, yet it is fraught with significant risks and challenges that can affect the quality, efficiency, and consistency of the final product. This study employed the Z-MABAC method within the ZE-numbers environment to systematically assess and rank the most critical failure modes in medicinal plant extraction. By integrating decision makers evaluations, fuzzy logic, and reliability assessments, the study provided a structured and data-driven approach for identifying key risk factors and prioritizing corrective actions.The findings revealed that sample-related issues, extraction method inefficiencies, and instrumental errors are among the most significant barriers to effective medicinal plant extraction. Sample variability and contamination (A2, A9, A10) were identified as top-ranked risks, highlighting the need for strict raw material selection, standardized handling procedures, and contamination control measures. Similarly, suboptimal extraction conditions (A6, A30) and instrumental errors (A5, A29) were found to be major contributors to process inefficiencies, leading to low extraction yields, degradation of bioactive compounds, and unreliable analytical results. These findings emphasize the necessity of process standardization, continuous equipment calibration, and optimization of extraction techniques to ensure high-quality outcomes. To mitigate these risks, it is crucial to implement best practices in sample preparation, optimize extraction parameters, and maintain rigorous quality control in instrumentation and data analysis. Additionally, training and skill development for personnel involved in the extraction process can significantly reduce human-related errors and enhance process consistency. The integration of advanced risk assessment models, such as the Z-MABAC method, offers a structured and reliable framework for improving decision-making in medicinal plant extraction.

Despite the advantages of the proposed ZE-number-based risk assessment framework, several limitations should be acknowledged. First, the methodology relies on expert judgments, and although fuzzy ZE-numbers are employed to capture uncertainty, reliability, and group consensus, the results may still be influenced by the subjective perceptions and experience of the selected decision-makers and experts. Second, the proposed framework involves multiple computational steps, including ZE-number construction, fuzzy transformation, and multi-criteria decision-making procedures, which may increase computational complexity and limit its direct applicability for large-scale problems with a high number of criteria and alternatives.

In addition, the proposed approach is demonstrated through a single case study in medicinal plant extraction; therefore, the generalizability of the results may be limited.

Future research should focus on expanding risk assessment frameworks by integrating machine learning and artificial intelligence (AI) algorithms to enhance real-time risk prediction and monitoring in medicinal plant extraction. Additionally, studies should explore sustainable and eco-friendly extraction techniques, such as green solvents and supercritical fluid extraction, to minimize environmental impact while maintaining high extraction efficiency. Further investigations into real-time quality control systems using spectroscopy and sensor-based technologies can improve process accuracy and reliability. Moreover, developing automated decision-making models that incorporate multiple MCDM techniques could enhance risk prioritization and process optimization, ultimately leading to safer, more effective, and standardized medicinal plant-derived products.
